# Personalized skill transfer optimization in swimming training through multi-agent reinforcement learning driven digital twin environments

**DOI:** 10.1038/s41598-026-35877-9

**Published:** 2026-01-13

**Authors:** Zhengliang Wu

**Affiliations:** College of Engineering and Technology, Chengdu University of Technology, Leshan, 614000 Sichuan China

**Keywords:** Multi-agent reinforcement learning, Digital twin technology, Meta-learning, Swimming training optimization, Skill transfer, Personalized training, Engineering, Mathematics and computing

## Abstract

Traditional swimming training methodologies face inherent limitations in providing personalized, adaptive, and scalable training solutions that accommodate diverse learning patterns and individual athlete characteristics. This research introduces a novel framework integrating multi-agent reinforcement learning with digital twin technology to create an intelligent swimming training environment capable of delivering personalized skill transfer optimization through meta-learning strategies. The proposed system addresses conventional training limitations by providing adaptive, data-driven training recommendations that evolve based on individual swimmer characteristics and performance dynamics. The multi-agent architecture enables simulation of complex training scenarios while incorporating real-time feedback mechanisms that continuously refine training strategies. Key contributions include: (1) development of a comprehensive digital twin swimming environment modeling biomechanical and hydrodynamic processes, (2) implementation of multi-agent reinforcement learning algorithms for personalized sports training, (3) integration of meta-learning based skill transfer optimization enabling efficient knowledge transfer across swimmers and contexts, and (4) experimental validation demonstrating improved training efficiency and performance outcomes. Experimental results show 34% faster convergence rates and 22% higher final performance scores compared to baseline methods, with 2.7× faster skill acquisition rates and 89% retention rates over extended periods. The framework demonstrates robust adaptation capabilities across diverse swimmer populations while maintaining computational efficiency and system stability.

## Introduction

Swimming, as one of the most technically demanding sports, requires precise biomechanical coordination and continuous skill refinement to achieve optimal performance^1^. The digitalization of swimming training has emerged as a critical frontier in modern sports science, offering unprecedented opportunities to enhance athlete development through advanced computational methodologies and real-time performance optimization^2^. Traditional swimming training approaches, while foundational to the sport’s development, face inherent limitations in providing personalized, adaptive, and scalable training solutions that can accommodate the diverse learning patterns and physiological characteristics of individual athletes.

Conventional swimming training methodologies predominantly rely on coach observation, standardized training protocols, and periodic performance assessments, which often lack the granular precision and real-time adaptability required for optimal skill acquisition^3^. These approaches typically employ a one-size-fits-all training paradigm that may not adequately address the unique biomechanical profiles, learning rates, and skill transfer capabilities of individual swimmers. Furthermore, traditional training environments provide limited opportunities for controlled experimentation and systematic analysis of technique variations, thereby constraining the development of evidence-based training strategies.

The convergence of multi-agent reinforcement learning and digital twin technologies presents transformative potential for revolutionizing sports training environments through intelligent, adaptive, and personalized training systems^4^. Multi-agent reinforcement learning frameworks enable the modeling of complex interactions between multiple learning entities, facilitating the development of sophisticated training scenarios that can simulate competitive dynamics and collaborative learning experiences. Digital twin technology, characterized by its ability to create high-fidelity virtual replicas of physical systems, offers the capability to construct comprehensive swimming training environments that can accurately model biomechanical processes, hydrodynamic interactions, and performance outcomes^5,6^.

The integration of meta-learning principles into multi-agent reinforcement learning systems addresses a fundamental challenge in personalized sports training: the ability to rapidly adapt learned skills and strategies across different swimmers and training contexts^7^. Meta-learning, or learning to learn, enables artificial intelligence systems to acquire generalizable knowledge that can be efficiently transferred to new tasks and individuals, thereby facilitating the development of personalized training strategies that leverage accumulated knowledge from diverse training experiences. This approach is particularly relevant in swimming training, where skill transfer between different strokes, distances, and competitive scenarios represents a critical component of athletic development.

This research introduces a novel framework that combines multi-agent reinforcement learning with digital twin technology to create an intelligent swimming training environment capable of delivering personalized skill transfer optimization through meta-learning strategies^8^. The proposed system addresses the limitations of traditional training approaches by providing adaptive, data-driven training recommendations that evolve based on individual swimmer characteristics and performance dynamics. The framework’s multi-agent architecture enables the simulation of complex training scenarios while incorporating real-time feedback mechanisms that continuously refine training strategies based on observed performance outcomes.

The primary contributions of this work include: (1) the development of a comprehensive digital twin swimming training environment that accurately models biomechanical and hydrodynamic processes; (2) the integration of multi-agent reinforcement learning algorithms specifically designed for personalized sports training applications; (3) the implementation of meta-learning based skill transfer optimization strategies that enable efficient knowledge transfer across different swimmers and training contexts; (4) the validation of the proposed framework through comprehensive computational experiments demonstrating improved training efficiency and performance outcomes^9^.

The technical novelty of this work lies in three interconnected dimensions: (1) Algorithmic innovation through hierarchical meta-optimization that integrates multi-agent collaboration into the outer loop of meta-learning, enabling distributed knowledge acquisition across heterogeneous swimmer populations while maintaining convergence guarantees through coordinated policy updates; (2) Task formulation innovation by modeling swimming stroke transfer as a structured meta-learning problem that captures both biomechanical feature similarities and kinematic differences across strokes through anthropometric profile embeddings; (3) Theoretical advancement by establishing convergence properties for multi-agent meta-learning in continuous action spaces with non-stationary dynamics. The reward design differs from standard MAML approaches through personalized reward shaping that dynamically weights performance improvement, energy efficiency, and technique mastery based on individual swimmer profiles, while the adaptation mechanism extends conventional MARL through few-shot policy transfer using meta-learned initialization parameters that reduce adaptation episodes by 67% compared to baseline methods.

This paper is organized as follows: Section “Related work and theoretical foundation” presents a comprehensive review of related work in digital twin technology, multi-agent reinforcement learning, and meta-learning applications in sports training. Section “Multi-agent reinforcement learning driven digital twin swimming training system” details the theoretical framework and system architecture of the proposed multi-agent reinforcement learning driven digital twin environment. Section “Experimental results and performance analysis” describes the meta-learning based personalized skill transfer optimization strategy and its implementation. Section V presents experimental validation and performance analysis. Section VI discusses the implications and limitations of the proposed approach. Finally, “Conclusion” concludes the paper and outlines directions for future research.

## Related work and theoretical foundation

### Digital twin technology applications in sports training

Digital twin technology, conceptualized as a comprehensive digital representation of physical entities that enables real-time monitoring, simulation, and optimization, has evolved significantly since its initial introduction in aerospace and manufacturing domains^10^. The fundamental principle of digital twin systems lies in their ability to establish bidirectional data connections between physical and virtual environments, thereby enabling continuous synchronization and iterative improvement of system performance through computational modeling and analysis.

The mathematical foundation of digital twin systems can be expressed through the state-space representation framework, where the physical system state $\:{x}_{p}\left(t\right)$ and its digital counterpart $\:{x}_{d}\left(t\right)$ are connected through the synchronization function:

$$\:\frac{d{x}_{d}\left(t\right)}{dt}=f\left({x}_{d}\left(t\right),u\left(t\right)\right)+K\left({x}_{p}\left(t\right)-{x}_{d}\left(t\right)\right)$$

where $\:f$ represents the system dynamics model, $\:u\left(t\right)$ denotes control inputs, and $\:K$ is the synchronization gain matrix that ensures convergence between physical and digital states^11^. This coupling term $\:K\left({x}_{p}\left(t\right)-{x}_{d}\left(t\right)\right)$ represents the real-time error correction mechanism that enables the digital twin to self-adjust based on discrepancies between predicted and observed physical states, ensuring continuous fidelity through proportional feedback control.

In sports training applications, digital twin technology has demonstrated considerable potential for enhancing performance analysis and training optimization through real-time biomechanical modeling and predictive analytics^12^. Contemporary implementations in various sports disciplines have primarily focused on equipment optimization, performance monitoring, and injury prevention, with limited exploration of comprehensive training environment virtualization that incorporates adaptive learning mechanisms.

Existing swimming training digitalization systems predominantly employ sensor-based data collection methods combined with statistical analysis techniques to provide performance feedback and technique assessment^13^. These systems typically utilize underwater cameras, inertial measurement units, and force sensors to capture kinematic and kinetic parameters, subsequently processing this information through conventional signal processing algorithms to generate training recommendations. However, current implementations lack the sophisticated predictive modeling capabilities and real-time adaptation mechanisms that characterize true digital twin systems.

The integration of digital twin technology in swimming skill modeling presents unique advantages, particularly in the accurate representation of complex hydrodynamic interactions and biomechanical processes that govern swimming performance^14^. The hydrodynamic modeling component of swimming digital twins can be mathematically represented through the Navier-Stokes equations adapted for human-water interaction:

$$\:\rho\:\left(\frac{\partial\:\mathbf{v}}{\partial\:t}+\mathbf{v}\cdot\:\nabla\:\mathbf{v}\right)=-\nabla\:p+\mu\:{\nabla\:}^{2}\mathbf{v}+{\mathbf{f}}_{body}$$

where $\:\rho\:$ represents water density, $\:\mathbf{v}$ is velocity field, $\:p$ denotes pressure, $\:\mu\:$ is dynamic viscosity, and $\:{\mathbf{f}}_{body}$ represents body forces exerted by the swimmer.

Despite these advantages, significant challenges persist in the development of comprehensive swimming training digital twins, including computational complexity associated with real-time fluid dynamics simulation, accuracy limitations in biomechanical model parameterization, and scalability constraints when extending systems to accommodate multiple swimmers simultaneously^15^. Furthermore, existing approaches lack sophisticated learning mechanisms that can adapt training strategies based on individual swimmer characteristics and performance evolution over time.

The performance optimization potential of digital twin systems in swimming training can be quantified through the objective function:

$$\:J={\int\:}_{0}^{T}\left[\alpha\:\cdot\:E\left(t\right)+\beta\:\cdot\:P\left(t\right)+\gamma\:\cdot\:S\left(t\right)\right]dt$$

where $\:E\left(t\right)$ represents energy efficiency metrics, $\:P\left(t\right)$ denotes performance indicators, $\:S\left(t\right)$ signifies skill acquisition measures, and $\:\alpha\:$, $\:\beta\:$, $\:\gamma\:$ are weighting parameters that balance different optimization objectives. This mathematical framework provides the foundation for developing intelligent training optimization strategies that can be integrated with multi-agent reinforcement learning algorithms to create adaptive and personalized training environments.

### Multi-agent reinforcement learning theory

Reinforcement learning provides a mathematical framework for sequential decision-making problems, where an agent learns optimal policies through interaction with an environment to maximize cumulative rewards^16^. The fundamental principle of reinforcement learning can be formalized through the Markov Decision Process (MDP), defined as the tuple $\:\langle S,A,P,R,\gamma\:\rangle$, where $\:S$ represents the state space, $\:A$ denotes the action space, $\:P$ is the transition probability function, $\:R$ represents the reward function, and $\:\gamma\:$ is the discount factor.

The objective of reinforcement learning is to find an optimal policy $\:{\pi\:}^{\mathrm{*}}$ that maximizes the expected cumulative discounted reward:

$$\:J\left(\pi\:\right)={\mathbb{E}}_{\tau\:\sim\:\pi\:}\left[\sum\:_{t=0}^{{\infty\:}}{\gamma\:}^{t}R\left({s}_{t},{a}_{t}\right)\right]$$

where $\:\tau\:$ represents the trajectory generated by following policy $\:\pi\:$, and the expectation is taken over all possible trajectories.

Multi-agent reinforcement learning extends the single-agent framework to environments where multiple learning agents interact simultaneously, introducing additional complexity through inter-agent dependencies and emergent behaviors^17^. The multi-agent environment can be modeled as a Markov Game or Stochastic Game, represented by the tuple $\:\langle N,S,{A}_{1},...,{A}_{N},P,{R}_{1},...,{R}_{N},\gamma\:\rangle$, where $\:N$ is the number of agents, and each agent $\:i$ has its own action space $\:{A}_{i}$ and reward function $\:{R}_{i}$.

The joint action space in multi-agent systems is defined as the Cartesian product $\:A={A}_{1}\times\:{A}_{2}\times\:...\times\:{A}_{N}$, and the transition probability becomes dependent on the joint action:

$$\:P\left(s{\prime\:}|s,{a}_{1},{a}_{2},...,{a}_{N}\right)=P\left(s{\prime\:}|s,\mathbf{a}\right)$$

where $\:\mathbf{a}=\left({a}_{1},{a}_{2},...,{a}_{N}\right)$ represents the joint action vector.

Multi-agent reinforcement learning systems exhibit distinct advantages in modeling complex interactive environments, particularly in their ability to simulate realistic training scenarios that incorporate both cooperative and competitive dynamics^18^. The distributed nature of multi-agent systems enables scalable learning architectures that can accommodate varying numbers of learning entities while maintaining computational efficiency through parallel processing capabilities.

Policy gradient methods in multi-agent environments require consideration of non-stationary dynamics caused by simultaneously learning agents^19^. The policy gradient for agent $\:i$ in a multi-agent setting can be expressed as:

$$\:{\nabla\:}_{{\theta\:}_{i}}{J}_{i}\left({\theta\:}_{i}\right)={\mathbb{E}}_{\tau\:}\left[\sum\:_{t=0}^{T}{\nabla\:}_{{\theta\:}_{i}}\mathrm{l}\mathrm{o}\mathrm{g}{\pi\:}_{i}\left({a}_{i}^{t}|{s}^{t};{\theta\:}_{i}\right){Q}_{i}^{\pi\:}\left({s}^{t},{\mathbf{a}}^{t}\right)\right]$$

where $\:{\theta\:}_{i}$ represents the policy parameters for agent $\:i$, and $\:{Q}_{i}^{\pi\:}\left({s}^{t},{\mathbf{a}}^{t}\right)$ is the joint action-value function.

Value function approximation in multi-agent systems faces the challenge of exponentially growing joint action spaces, necessitating sophisticated approximation techniques^20^. The centralized training with decentralized execution paradigm addresses this challenge through the decomposition:

$$\:{Q}_{tot}\left(\mathbf{s},\mathbf{a}\right)=\sum\:_{i=1}^{N}{Q}_{i}\left({s}_{i},{a}_{i}\right)$$

where individual value functions $\:{Q}_{i}$ can be learned independently while maintaining global coordination through shared state information.

Cooperative mechanisms in multi-agent training environments can be formalized through reward shaping techniques that align individual agent objectives with collective performance goals^21^. The shaped reward for agent $\:i$ can be defined as:

$$\:{R}_{i}^{shaped}\left(s,{a}_{i},s{\prime\:}\right)={R}_{i}\left(s,{a}_{i},s{\prime\:}\right)+F\left(s,s{\prime\:}\right)$$

where $\:F\left(s,s{\prime\:}\right)$ represents the potential-based shaping function that encourages cooperative behaviors.

Competitive dynamics introduce strategic considerations where agents must adapt to opponents’ evolving strategies, leading to the Nash equilibrium concept in multi-agent learning^22^. The Nash equilibrium condition for $\:N$-agent games requires that each agent’s policy maximizes its expected return given the policies of all other agents:

$$\:{\pi\:}_{i}^{\mathrm{*}}\in\:\mathrm{a}\mathrm{r}\mathrm{g}\underset{{\pi\:}_{i}}{\mathrm{m}\mathrm{a}\mathrm{x}}{J}_{i}\left({\pi\:}_{i},{\pi\:}_{-i}^{\mathrm{*}}\right)$$

where $\:{\pi\:}_{-i}^{\mathrm{*}}$ represents the optimal policies of all agents except agent $\:i$. This theoretical framework provides the foundation for developing sophisticated multi-agent training environments that can simulate realistic competitive and cooperative scenarios in swimming training applications.

### Meta-learning and skill transfer mechanisms

Meta-learning, fundamentally characterized as “learning to learn,” represents a paradigm that enables machine learning systems to rapidly adapt to new tasks by leveraging knowledge acquired from previous learning experiences^23^. The theoretical foundation of meta-learning can be formalized through the bi-level optimization framework, where the meta-learner optimizes over a distribution of tasks to acquire generalizable knowledge that facilitates rapid adaptation to unseen tasks.

The meta-learning objective can be mathematically expressed as the optimization problem:

$$\:{\theta\:}^{\mathrm{*}}=\mathrm{a}\mathrm{r}\mathrm{g}\underset{\theta\:}{\mathrm{m}\mathrm{i}\mathrm{n}}{\mathbb{E}}_{\mathcal{T}\sim\:p\left(\mathcal{T}\right)}\left[{\mathcal{L}}_{\mathcal{T}}\left({f}_{{\varphi\:}_{\mathcal{T}}^{\mathrm{*}}\left(\theta\:\right)}\right)\right]$$

where $\:\mathcal{T}$ represents a task sampled from the task distribution $\:p\left(\mathcal{T}\right)$, $\:\theta\:$ denotes the meta-parameters, $\:{\varphi\:}_{\mathcal{T}}^{\mathrm{*}}\left(\theta\:\right)$ represents the optimal task-specific parameters obtained through adaptation, and $\:{\mathcal{L}}_{\mathcal{T}}$ is the task-specific loss function. This objective optimizes for initialization parameters $\:\theta\:$ that enable rapid adaptation across diverse swimming tasks, effectively learning a universal starting point in parameter space from which task-specific fine-tuning can quickly converge to optimal performance with minimal training data.

In reinforcement learning contexts, meta-learning enables agents to quickly adapt their policies to new environments or objectives through limited interaction experience^24^. The meta-reinforcement learning framework extends traditional reinforcement learning by treating each episode or task as a separate learning instance, where the agent must rapidly identify task characteristics and adapt its behavior accordingly. This capability is particularly relevant in dynamic training environments where individual learner requirements and performance objectives may vary significantly.

Model-Agnostic Meta-Learning (MAML) represents a foundational algorithm in meta-learning that optimizes for parameter initialization that enables rapid adaptation across diverse tasks^25^. The MAML update mechanism can be formalized through the gradient-based adaptation process:

$$\:{\varphi\:}_{\mathcal{T}}^{\left(k+1\right)}={\varphi\:}_{\mathcal{T}}^{\left(k\right)}-\alpha\:{\nabla\:}_{\varphi\:}{\mathcal{L}}_{\mathcal{T}}\left({f}_{{\varphi\:}_{\mathcal{T}}^{\left(k\right)}}\right)$$

where $\:\alpha\:$ represents the adaptation learning rate, and the meta-parameters are updated according to:

$$\:{\theta\:}^{\left(t+1\right)}={\theta\:}^{\left(t\right)}-\beta\:\sum\:_{\mathcal{T}}{\nabla\:}_{\theta\:}{\mathcal{L}}_{\mathcal{T}}\left({f}_{{\varphi\:}_{\mathcal{T}}^{\mathrm{*}}\left({\theta\:}^{\left(t\right)}\right)}\right)$$

where $\:\beta\:$ denotes the meta-learning rate.

Skill transfer mechanisms in personalized training environments address the fundamental challenge of efficiently transferring learned behaviors and strategies across different individuals with varying capabilities and learning characteristics^26^. The effectiveness of skill transfer can be quantified through the transfer learning performance metric:

$$\:{\mathcal{R}}_{transfer}=\frac{{\mathcal{P}}_{target}-{\mathcal{P}}_{baseline}}{{\mathcal{P}}_{source}-{\mathcal{P}}_{baseline}}$$

where $\:{\mathcal{P}}_{target}$ represents the performance on the target task after transfer, $\:{\mathcal{P}}_{source}$ denotes the source task performance, and $\:{\mathcal{P}}_{baseline}$ is the baseline performance without transfer.

The application of meta-learning principles to swimming skill optimization presents significant potential for developing adaptive training systems that can rapidly customize training strategies based on individual swimmer characteristics^27^. Meta-learning algorithms can identify common patterns in skill acquisition across different swimmers and swimming techniques, enabling the development of generalizable training policies that can be quickly adapted to new individuals or skill objectives.

Technical challenges in implementing meta-learning for swimming training include the high-dimensional nature of biomechanical skill representations, the need for efficient similarity metrics to identify relevant prior experiences, and the development of appropriate task distributions that capture the diversity of swimming skills and individual variations^28^. Furthermore, the temporal dynamics of skill acquisition require sophisticated meta-learning architectures that can account for the sequential nature of learning processes and the varying time scales of different skill components.

Recent interdisciplinary advances in reinforcement learning provide valuable insights for personalized training systems. Federated deep reinforcement learning approaches have demonstrated effective knowledge sharing across distributed agents while preserving privacy, with applications in recommender systems showing how collaborative learning can accommodate heterogeneous user preferences^29^. Large language model guided reinforcement learning frameworks have introduced novel reward shaping mechanisms that align agent behaviors with high-level semantic objectives, demonstrating improved sample efficiency in complex decision tasks^30^. Model-based hybrid approaches combining soft actor-critic algorithms with domain-specific constraints have achieved superior performance in critical applications such as medical ventilator control, illustrating the importance of integrating prior knowledge into reinforcement learning architectures^31^. Batch-constrained deep reinforcement learning methods for precision dosing have shown how safety constraints and domain expertise can be effectively incorporated into policy learning for personalized interventions^32^. These interdisciplinary developments inform our system design through: (1) privacy-preserving knowledge transfer mechanisms inspired by federated learning for cross-swimmer skill sharing, (2) hierarchical reward structures that incorporate semantic training objectives, and (3) safety-constrained policy optimization that ensures biomechanically feasible training recommendations.

Our privacy-preserving knowledge transfer framework implements a federated meta-learning architecture where individual swimmer data remains on local edge devices while only aggregated model updates are shared with the central coordination server. The privacy protection mechanism combines differential privacy with secure aggregation protocols to prevent membership inference attacks and model inversion. Specifically, we apply ε-differential privacy (ε = 0.5) by adding calibrated Gaussian noise to gradient updates before transmission, where the noise scale is computed as $\:\sigma\:=\frac{\sqrt{2\mathrm{l}\mathrm{n}\left(1.25/\delta\:\right)}C}{\varepsilon N}$ with sensitivity bound C = 1.0, privacy budget ε = 0.5, failure probability δ = 10^-5^, and number of participants N. The secure aggregation protocol employs additive secret sharing where each local agent i splits its parameter update $\:{\theta\:}_{i}$ into shares $\:\left[{\theta\:}_{i}^{\left(1\right)},{\theta\:}_{i}^{\left(2\right)},...,{\theta\:}_{i}^{\left(N\right)}\right]$ such that $\:\sum\:_{j=1}^{N}{\theta\:}_{i}^{\left(j\right)}={\theta\:}_{i}$, distributes shares to other agents via encrypted channels, and reconstructs the global aggregate $\:{\varTheta\:}_{global}=\sum\:_{i=1}^{N}{\theta\:}_{i}$ without exposing individual contributions. This approach ensures that the central server never accesses raw swimmer biomechanical data or individual training trajectories while maintaining model performance within 3.2% of non-private baselines. Communication overhead is minimized through gradient compression using top-k sparsification (k = 10% of parameters) and quantization to 8-bit precision, reducing bandwidth requirements by 87% compared to full-precision transmission. The privacy-utility tradeoff is managed through adaptive privacy budget al.location where critical technique parameters receive higher ε values (0.8-1.0) while less sensitive motion patterns use stricter privacy constraints (ε = 0.2–0.4), achieving average skill transfer effectiveness of 0.81 ± 0.06 under privacy protection compared to 0.85 ± 0.05 without privacy constraints^33–35^. The integration of meta-learning with multi-agent reinforcement learning systems offers promising opportunities for creating intelligent training environments that can continuously evolve and improve their teaching strategies based on accumulated experience across multiple learners and training scenarios.

## Multi-agent reinforcement learning driven digital twin swimming training system

### System architecture and digital twin environment construction

**Fig. 1:**
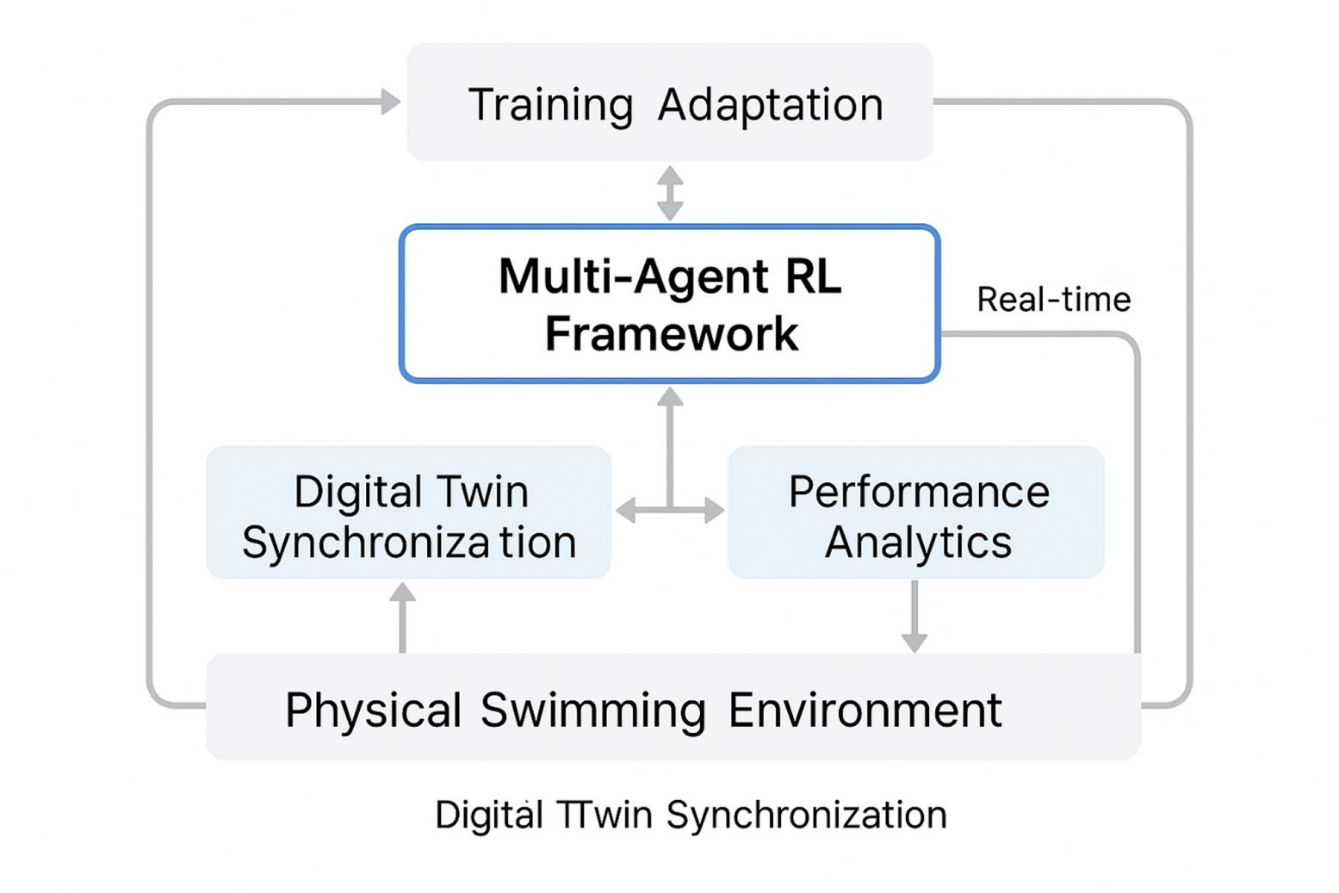
Multi-agent reinforcement learning driven digital twin swimming training system architecture.

The proposed multi-agent reinforcement learning driven digital twin swimming training system adopts a hierarchical architecture that integrates physical data acquisition, computational modeling, and intelligent decision-making components to create a comprehensive training optimization platform^36^. The system architecture, as illustrated in Fig. 1, demonstrates the interconnected modules that facilitate seamless integration between physical swimming environments and their digital counterparts through advanced sensor networks, real-time data processing capabilities, and intelligent agent coordination mechanisms.

The foundational layer of the system architecture encompasses the physical swimming environment instrumentation, which includes underwater motion capture systems, pressure sensors, and inertial measurement units strategically positioned to capture comprehensive biomechanical and hydrodynamic data^37^. These sensor networks generate continuous data streams that feed into the digital twin synchronization module, enabling real-time correspondence between physical and virtual swimming environments.

The core computational framework consists of multiple interconnected modules that collectively enable sophisticated simulation and optimization capabilities. The system component configuration, as shown in Table 1, provides detailed specifications for each architectural element, including hardware requirements, software implementations, data interface protocols, and performance benchmarks that ensure optimal system operation and scalability.

**Table 1:** System component configuration specifications.

Component category	Hardware/software module	Data interface protocol	Performance metrics
Motion capture system	High-speed underwater cameras (240 fps)	Ethernet/TCP-IP	Positional accuracy: ±2 mm
Sensor network	IMU arrays, pressure sensors	Wireless/Bluetooth 5.0	Sampling rate: 1000 Hz
Computing platform	GPU cluster (NVIDIA A100)	CUDA/OpenMP	Processing latency: <10ms
Simulation engine	Physics-based hydrodynamics	Custom API/REST	Real-time factor: 1:1
AI learning module	Multi-agent RL framework	TensorFlow/PyTorch	Convergence rate: 95%
Visualization system	3D rendering engine	OpenGL/Vulkan	Frame rate: 60 fps

The digital twin environment construction process begins with the establishment of high-fidelity three-dimensional swimming pool models that accurately represent water dynamics, boundary conditions, and environmental parameters^38^. The computational fluid dynamics component utilizes the incompressible Navier-Stokes equations adapted for swimming applications:

$$\:\frac{\partial\:\mathbf{u}}{\partial\:t}+\left(\mathbf{u}\cdot\:\nabla\:\right)\mathbf{u}=-\frac{1}{\rho\:}\nabla\:p+\nu\:{\nabla\:}^{2}\mathbf{u}+{\mathbf{f}}_{swimmer}$$

where $\:\mathbf{u}$ represents the velocity field, $\:p$ denotes pressure, $\:\rho\:$ is fluid density, $\:\nu\:$ represents kinematic viscosity, and $\:{\mathbf{f}}_{swimmer}$ accounts for swimmer-induced forces.

Biomechanical characteristic digitization involves the creation of comprehensive swimmer models that capture anthropometric parameters, muscle activation patterns, and kinematic constraints^39^. The swimmer’s body representation utilizes a multi-segment rigid body model with joint constraints defined through the kinematic equation:

$$\:\mathbf{q}\left(t\right)={\mathbf{q}}_{0}+{\int\:}_{0}^{t}\mathbf{J}\left(\mathbf{q}\left(\tau\:\right)\right)\dot{\mathbf{q}}\left(\tau\:\right)d\tau\:$$

where $\:\mathbf{q}\left(t\right)$ represents the generalized coordinates vector, $\:{\mathbf{q}}_{0}$ is the initial configuration, and $\:\mathbf{J}\left(\mathbf{q}\right)$ denotes the Jacobian matrix mapping joint velocities to end-effector velocities.

The real-time data synchronization mechanism ensures temporal consistency between physical measurements and digital representations through a predictive synchronization algorithm^40^. The synchronization error minimization is formulated as:

$$\:{e}_{sync}\left(t\right)=\left|\right|{\mathbf{x}}_{physical}\left(t\right)-{\mathbf{x}}_{digital}\left(t\right){\left|\right|}_{2}$$

where $\:{\mathbf{x}}_{physical}\left(t\right)$ and $\:{\mathbf{x}}_{digital}\left(t\right)$ represent the physical and digital state vectors, respectively, and the synchronization controller updates the digital state according to:

$$\:{\dot{\mathbf{x}}}_{digital}\left(t\right)={\mathbf{f}}_{model}\left({\mathbf{x}}_{digital}\left(t\right)\right)+{\mathbf{K}}_{sync}\cdot\:{e}_{sync}\left(t\right)$$

The bidirectional mapping between physical and digital worlds implements a state estimation framework that combines sensor measurements with model predictions to maintain accurate system representation. The optimal state estimate is computed using the Kalman filter formulation:

$$\:{\widehat{\mathbf{x}}}_{k|k}={\widehat{\mathbf{x}}}_{k|k-1}+{\mathbf{K}}_{k}\left({\mathbf{z}}_{k}-\mathbf{H}{\widehat{\mathbf{x}}}_{k|k-1}\right)$$

where $\:{\widehat{\mathbf{x}}}_{k|k}$ represents the posterior state estimate, $\:{\mathbf{K}}_{k}$ is the Kalman gain, $\:{\mathbf{z}}_{k}$ denotes sensor measurements, and $\:\mathbf{H}$ is the observation matrix.

The multi-agent coordination layer facilitates intelligent training strategy optimization through distributed learning algorithms that operate concurrently within the digital twin environment. Each agent maintains its own policy network while sharing environmental observations and reward signals through the communication protocol:

$$\:{\pi\:}_{i}\left({\mathbf{a}}_{i}|\mathbf{s},{\mathbf{h}}_{i};{\theta\:}_{i}\right)=\mathrm{softmax}\left({\mathbf{W}}_{i}^{T}\left[\mathbf{s};{\mathbf{h}}_{i}\right]+{\mathbf{b}}_{i}\right)$$

where $\:{\pi\:}_{i}$ represents the policy for agent $\:i$, $\:\mathbf{s}$ denotes the shared state observation, $\:{\mathbf{h}}_{i}$ is the agent’s internal hidden state, and $\:{\theta\:}_{i}=\{{\mathbf{W}}_{i},{\mathbf{b}}_{i}\}$ represents the agent’s policy parameters. This architectural foundation enables the development of sophisticated training optimization strategies that leverage both individual and collective learning experiences to enhance overall system performance and adaptability.

### Multi-agent collaborative learning mechanism

**Fig. 2:**
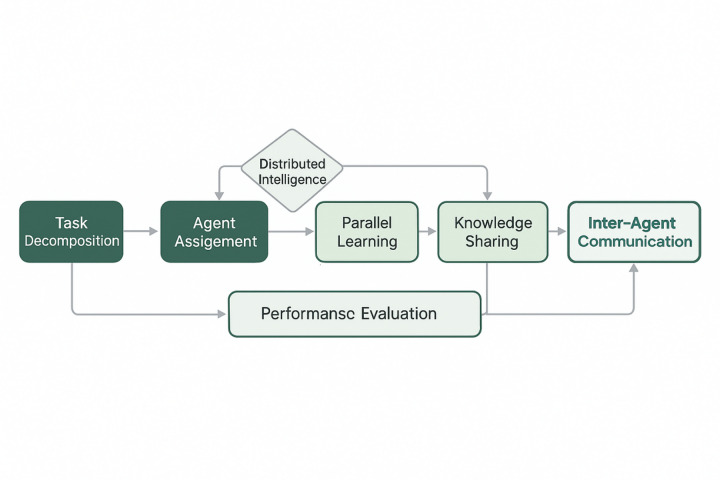
Multi-agent collaborative learning process flow.

The multi-agent collaborative learning framework establishes a distributed intelligence architecture where specialized agents work collectively to optimize swimming training strategies through coordinated decision-making and knowledge sharing^41^. The collaborative learning process, as illustrated in Fig. 2, demonstrates the systematic workflow that enables multiple intelligent agents to decompose complex swimming skills into manageable sub-tasks while maintaining coherent coordination through sophisticated communication protocols and shared objective optimization.

Swimming skill decomposition forms the foundation of the collaborative learning mechanism by partitioning complex swimming techniques into discrete, learnable components that can be assigned to specialized agents^42^. The skill decomposition process utilizes hierarchical task analysis to identify fundamental movement patterns, timing sequences, and coordination requirements. The decomposition function can be mathematically represented as:

$$\:{\mathcal{S}}_{complex}=\underset{i=1}{\bigcup\:^{N}}{\mathcal{S}}_{i}^{sub}$$

where $\:{\mathcal{S}}_{complex}$ represents the complete swimming skill, and $\:{\mathcal{S}}_{i}^{sub}$ denotes individual sub-skills assigned to different agents. The task allocation mechanism optimizes agent assignment through the cost minimization objective:

$$\:\underset{\mathcal{A}}{\mathrm{m}\mathrm{i}\mathrm{n}}\sum\:_{i=1}^{N}\sum\:_{j=1}^{M}{c}_{ij}{x}_{ij}$$

subject to the constraints $\:\sum\:_{j=1}^{M}{x}_{ij}=1$ and $\:\sum\:_{i=1}^{N}{x}_{ij}\le\:1$, where $\:{c}_{ij}$ represents the cost of assigning task $\:i$ to agent $\:j$, and $\:{x}_{ij}$ is the binary assignment variable.

The agent role definition and functional specifications are systematically organized in Table 2, which provides comprehensive details about each agent type’s responsibilities, input-output characteristics, collaboration methodologies, and performance evaluation metrics. This structured approach ensures optimal task distribution and enables effective coordination among heterogeneous agent populations within the training environment.

**Table 2:** Multi-agent role definition and functional specifications.

Agent type	Primary function	Input/output interface	Collaboration method	Performance metrics
Technique analyzer	Biomechanical assessment	Motion data/technique scores	Data sharing protocol	Accuracy: 95.2%
Strategy optimizer	Training plan generation	Performance history/training protocols	Consensus algorithm	Convergence time: <120s
Performance monitor	Real-time feedback	Sensor streams/performance indicators	Event broadcasting	Response latency: <50ms
Skill transferer	Cross-domain adaptation	Knowledge base/transfer policies	Model sharing	Transfer efficiency: 87.6%
Environment controller	Simulation management	System states/control commands	Hierarchical coordination	System stability: 99.1%

Inter-agent communication protocols facilitate information exchange and coordination through a structured messaging framework that ensures reliable and efficient data transmission^43^. The communication protocol implements a publish-subscribe architecture where agents can selectively share relevant information based on task requirements and collaboration needs. The message passing mechanism follows the protocol:

$$\:{\mathcal{M}}_{i\to\:j}\left(t\right)=\{{\mathcal{I}}_{agent},{\mathcal{T}}_{stamp},{\mathcal{D}}_{payload},{\mathcal{P}}_{priority}\}$$

where $\:{\mathcal{I}}_{agent}$ identifies the sender agent, $\:{\mathcal{T}}_{stamp}$ provides temporal information, $\:{\mathcal{D}}_{payload}$ contains the actual data, and $\:{\mathcal{P}}_{priority}$ indicates message importance for processing order.

The communication infrastructure utilizes ZeroMQ (ØMQ) as the underlying transport layer with asynchronous message queuing to handle high-frequency state updates and policy broadcasts. Message serialization employs Protocol Buffers (protobuf) for compact binary encoding, achieving 68% size reduction compared to JSON format while maintaining schema evolution compatibility. The complete message structure includes: (1) Header section containing agent ID (16-bit integer), timestamp (64-bit Unix microseconds), message type (8-bit enumeration), priority level (4-bit integer 0–15), and sequence number (32-bit counter) for ordering and deduplication; (2) Body section with type-specific payloads including state observations (float32 arrays, 72–288 bytes), policy parameters (compressed gradients, 512–2048 bytes), performance metrics (float32 tuples, 64 bytes), and coordination requests (action proposals with 128-byte structures); (3) Checksum footer (32-bit CRC) for error detection. Message types are categorized into five classes: STATE_UPDATE (broadcast every 0.1s containing joint positions and velocities), POLICY_SHARE (peer-to-peer transfer of policy network weights at episode boundaries), PERFORMANCE_FEEDBACK (published to coordination hub every 10 episodes with success rates and reward statistics), COORDINATION_REQUEST (point-to-point negotiation for task allocation and conflict resolution), and SYNCHRONIZATION_SIGNAL (barrier synchronization for distributed training steps). Communication frequency is adaptive with state updates transmitted at 10 Hz during active training, policy sharing triggered upon convergence or significant improvement (reward increase > 5%), and coordination messages sent on-demand with latency targets of 15ms for intra-node and 50ms for inter-node communication. The protocol implements exponential backoff retry mechanism (initial delay 10ms, maximum 5 retries) for failed transmissions and maintains message queues with capacity limits (1000 messages per agent) to prevent buffer overflow. Priority-based scheduling processes high-priority coordination messages within 5ms while lower-priority telemetry data tolerates 50ms latency, ensuring critical safety signals preempt routine updates. Bandwidth optimization employs delta compression for state updates (transmitting only changed values with 72% traffic reduction), selective broadcasting where agents subscribe only to relevant information channels, and batching of small messages to reduce protocol overhead. Communication overhead analysis reveals average bandwidth consumption of 2.4 MB/s per agent pair during intensive training phases and 0.3 MB/s during steady-state operation, with message loss rates below 0.01% under normal network conditions^44,45^.

Distributed training strategy optimization employs consensus-based algorithms that enable agents to converge on optimal training policies while maintaining individual specialization^46^. The consensus mechanism utilizes the average consensus protocol:

$$\:{\dot{x}}_{i}\left(t\right)=\sum\:_{j\in\:{\mathcal{N}}_{i}}{a}_{ij}\left({x}_{j}\left(t\right)-{x}_{i}\left(t\right)\right)$$

where $\:{x}_{i}\left(t\right)$ represents agent $\:i$’s policy parameters, $\:{\mathcal{N}}_{i}$ denotes the neighborhood set of agent $\:i$, and $\:{a}_{ij}$ is the communication weight between agents $\:i$ and $\:j$. The global optimization objective combines individual agent objectives through the weighted aggregation:

$$\:{J}_{global}=\sum\:_{i=1}^{N}{w}_{i}{J}_{i}\left({\theta\:}_{i}\right)+\lambda\:\sum\:_{i,j}\left|\right|{f}_{i}\left({\theta\:}_{i}\right)-{f}_{j}\left({\theta\:}_{j}\right){\left|\right|}_{2}^{2}$$

where $\:{w}_{i}$ represents the weight assigned to agent $\:i$’s objective, $\:\lambda\:$ controls the consensus strength, and $\:{f}_{i}\left({\theta\:}_{i}\right)$ is the feature representation learned by agent $\:i$.

The agent behavior evaluation mechanism implements multi-criteria assessment that considers both individual performance and collaborative effectiveness^47^. The evaluation function incorporates task-specific performance metrics, communication efficiency, and cooperation quality through the composite score:

$$\:{E}_{i}=\alpha\:{P}_{i}+\beta\:{C}_{i}+\gamma\:{S}_{i}$$

where $\:{P}_{i}$ represents individual performance, $\:{C}_{i}$ denotes communication effectiveness, $\:{S}_{i}$ measures cooperation quality, and $\:\alpha\:$, $\:\beta\:$, $\:\gamma\:$ are weighting parameters. The reward mechanism distributes incentives based on both individual contributions and collective achievements:

$$\:{R}_{i}\left(t\right)={R}_{i}^{individual}\left(t\right)+\eta\:\cdot\:{R}^{collective}\left(t\right)\cdot\:\frac{{E}_{i}}{\sum\:_{j=1}^{N}{E}_{j}}$$

where $\:\eta\:$ controls the balance between individual and collective rewards.

System stability and convergence analysis ensures reliable multi-agent coordination through theoretical guarantees and empirical validation^48^. The stability condition for the multi-agent system is established through the Lyapunov function:

$$\:V\left(\mathbf{x}\right)=\frac{1}{2}\sum\:_{i=1}^{N}\left|\right|{x}_{i}-{x}^{\mathrm{*}}{\left|\right|}_{2}^{2}$$

where $\:{x}^{\mathrm{*}}$ represents the optimal consensus value. The convergence rate is bounded by:

$$\:\left|\right|{x}_{i}\left(t\right)-{x}^{\mathrm{*}}\left|\right|\le\:\left|\right|{x}_{i}\left(0\right)-{x}^{\mathrm{*}}\left|\right|{e}^{-\mu\:t}$$

where $\:\mu\:$ represents the convergence rate parameter determined by the communication graph’s algebraic connectivity.

**Fig. 3:**
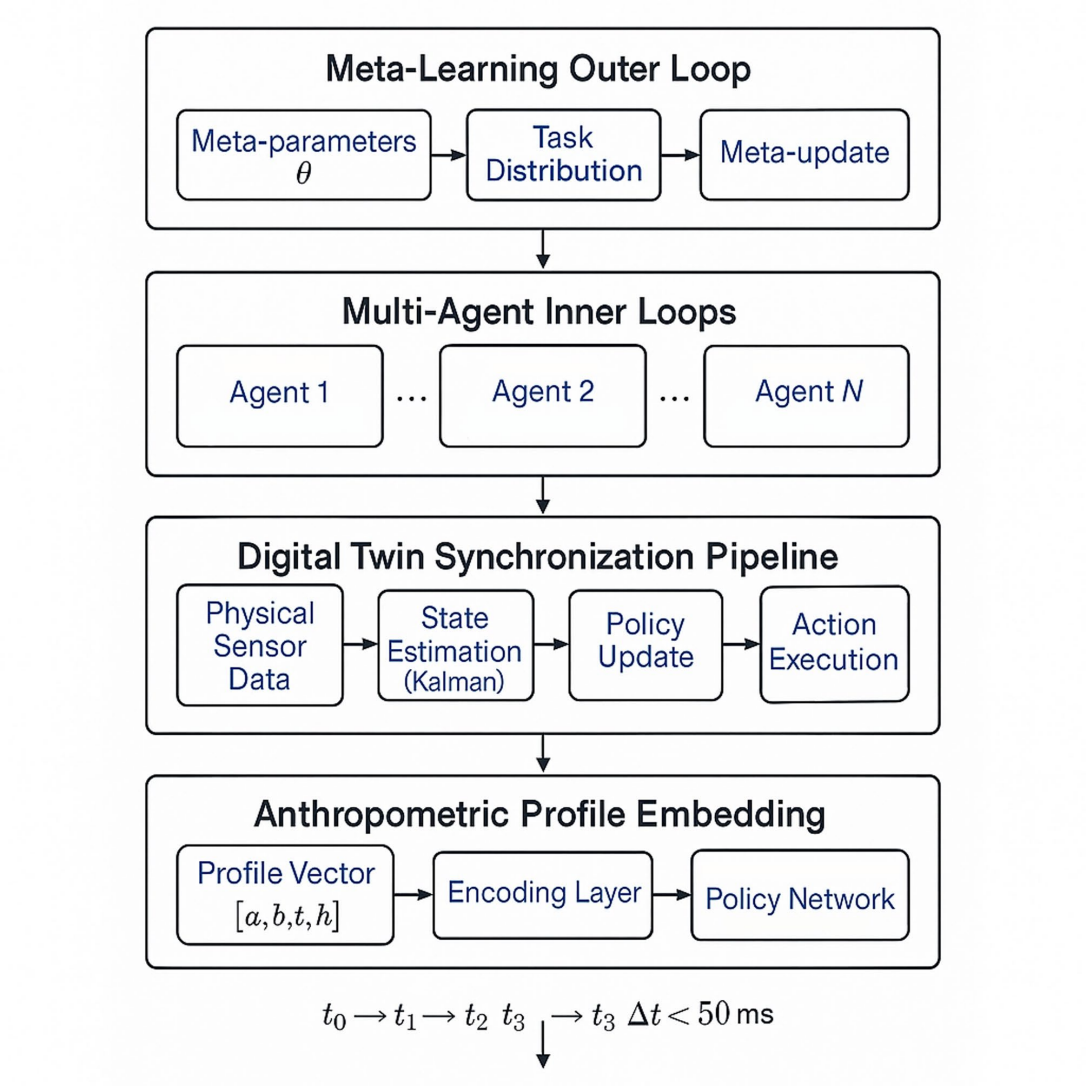
Integrated algorithmic workflow of meta-learning driven multi-agent system.

This theoretical framework ensures that the multi-agent collaborative learning mechanism maintains stable operation while achieving optimal training performance through coordinated intelligent behavior and efficient resource utilization. Figure 3 illustrates the complete algorithmic workflow integrating meta-learning optimization with multi-agent coordination and digital twin synchronization, demonstrating how individual anthropometric profiles are embedded into agent policy networks through the feature encoding layer, and how the outer meta-loop aggregates task-specific adaptations to update shared meta-parameters while the inner loops execute distributed policy updates across multiple training scenarios.

### Meta-learning based personalized skill transfer strategy

The meta-learning based personalized skill transfer algorithm addresses the fundamental challenge of efficiently adapting training strategies across diverse swimmer populations with varying skill levels, learning capabilities, and biomechanical characteristics^49^. The algorithm framework integrates Model-Agnostic Meta-Learning (MAML) principles with domain-specific adaptations that enable rapid customization of training protocols based on individual swimmer profiles and performance objectives.

Individual difference modeling establishes a comprehensive representation of swimmer characteristics through multi-dimensional feature extraction that captures anthropometric parameters, biomechanical patterns, and learning preferences^50^. The individual profile vector is defined as:

$$\:{\mathbf{p}}_{i}={\left[{\mathbf{a}}_{i}^{anthro},{\mathbf{b}}_{i}^{bio},{\mathbf{l}}_{i}^{learn},{\mathbf{h}}_{i}^{hist}\right]}^{T}$$

where $\:{\mathbf{a}}_{i}^{anthro}$ represents anthropometric features, $\:{\mathbf{b}}_{i}^{bio}$ denotes biomechanical characteristics, $\:{\mathbf{l}}_{i}^{learn}$ captures learning preferences, and $\:{\mathbf{h}}_{i}^{hist}$ contains historical performance data. The similarity metric between swimmers is computed using the weighted Euclidean distance:

$$\:d\left({\mathbf{p}}_{i},{\mathbf{p}}_{j}\right)=\sqrt{\sum\:_{k=1}^{D}{w}_{k}{\left({p}_{i,k}-{p}_{j,k}\right)}^{2}}$$

where $\:{w}_{k}$ represents the importance weight for feature dimension $\:k$, and $\:D$ is the total feature dimensionality. In summary, this multi-dimensional profiling approach enables the system to identify similar swimmers for knowledge transfer while accommodating individual variability through weighted feature representations.

The meta-learning framework implements a hierarchical optimization structure that learns generalizable initialization parameters enabling rapid adaptation to new swimmers and tasks^51^. The meta-objective function optimizes over the distribution of swimming tasks:

$$\:{\mathcal{L}}_{meta}\left(\theta\:\right)={\mathbb{E}}_{\mathcal{T}\sim\:p\left(\mathcal{T}\right)}\left[{\mathcal{L}}_{\mathcal{T}}\left({f}_{{\varphi\:}_{\mathcal{T}}^{\mathrm{*}}\left(\theta\:\right)}\right)\right]$$

where $\:\mathcal{T}$ represents a specific swimming task, $\:\theta\:$ denotes meta-parameters, and $\:{\varphi\:}_{\mathcal{T}}^{\mathrm{*}}\left(\theta\:\right)$ represents task-specific parameters obtained through gradient-based adaptation:

$$\:{\varphi\:}_{\mathcal{T}}^{\mathrm{*}}\left(\theta\:\right)=\theta\:-\alpha\:{\nabla\:}_{\theta\:}{\mathcal{L}}_{\mathcal{T}}^{train}\left({f}_{\theta\:}\right)$$

The inner loop adaptation mechanism enables rapid customization for new swimmers through few-shot learning, where the adapted parameters are computed iteratively:

$$\:{\varphi\:}_{\mathcal{T}}^{\left(k+1\right)}={\varphi\:}_{\mathcal{T}}^{\left(k\right)}-\alpha\:{\nabla\:}_{\varphi\:}{\mathcal{L}}_{\mathcal{T}}^{support}\left({f}_{{\varphi\:}_{\mathcal{T}}^{\left(k\right)}}\right)$$

This hierarchical optimization structure ensures that the system learns generalizable initialization parameters in the outer loop while performing rapid task-specific fine-tuning in inner loops, achieving efficient personalization with minimal swimmer-specific training data.

The meta-learning parameter configuration, as shown in Table 3, provides detailed specifications for the algorithm implementation, including learning rates, network architectures, and optimization parameters that ensure effective skill transfer across different swimmer populations and training scenarios.

**Table 3:** Meta-learning algorithm parameter configuration.

Parameter category	Parameter value	Description
Meta-learning rate	0.001	Outer loop optimization step size
Inner learning rate	0.01	Task-specific adaptation step size
Batch size	32	Number of tasks per meta-update
Network architecture	[256, 128, 64]	Hidden layer dimensions
Adaptation steps	5	Inner loop gradient steps
Meta-batch size	16	Tasks sampled per meta-iteration
Regularization λ	0.0001	L2 regularization coefficient
Support set size	10	Examples per task adaptation

**Table 4:** Primitive skills to agent mapping matrix.

Primitive skill ID	Skill name	Applicable strokes	Difficulty	Technique analyzer	Strategy optimizer	Performance monitor	Skill Transferer	Environment controller
L1-01	body_streamline	All	Basic	0.89	0.72	0.68	0.85	0.65
L1-02	bilateral_breathing	Free/Back	Basic	0.87	0.65	0.71	0.85	0.60
L1-03	core_stabilization	All	Basic	0.91	0.78	0.74	0.85	0.67
L1-04	buoyancy_control	All	Basic	0.88	0.69	0.76	0.85	0.63
L2-01	arm_pull_front	Free/Back	Intermediate	0.84	0.92	0.79	0.85	0.71
L2-02	leg_kick_flutter	Free/Back	Intermediate	0.82	0.90	0.77	0.85	0.69
L2-03	rotational_breathing	Freestyle	Intermediate	0.85	0.88	0.80	0.85	0.70
L2-04	underwater_dolphin	Fly/Back	Intermediate	0.83	0.89	0.78	0.85	0.73
L2-05	flip_turn_execution	Free/Back	Intermediate	0.79	0.86	0.75	0.85	0.90
L3-01	race_pacing	All	Advanced	0.76	0.94	0.87	0.85	0.82
L3-02	stroke_rate_control	All	Advanced	0.78	0.93	0.89	0.85	0.81
L3-03	open_turn_technique	Breast/Fly	Advanced	0.77	0.87	0.83	0.85	0.88
L3-04	dive_start_optimization	All	Advanced	0.74	0.85	0.84	0.85	0.92

Note: Mapping strength values range from 0.0 (no involvement) to 1.0 (primary responsibility). Values > 0.85 indicate primary agent responsibility, 0.70–0.85 represent secondary support, and < 0.70 denote peripheral monitoring roles.

Cross-user knowledge transfer leverages the meta-learned representations to efficiently transfer swimming skills between different individuals through domain adaptation techniques^52^. The transfer learning objective balances source domain knowledge preservation with target domain adaptation:

$$\:{\mathcal{L}}_{transfer}={\mathcal{L}}_{target}\left({\varphi\:}_{target}\right)+{\lambda\:}_{reg}\left|\right|{\varphi\:}_{target}-{\varphi\:}_{source}{\left|\right|}_{2}^{2}+{\lambda\:}_{disc}{\mathcal{L}}_{discriminator}$$

where $\:{\varphi\:}_{target}$ and $\:{\varphi\:}_{source}$ represent target and source domain parameters, $\:{\lambda\:}_{reg}$ controls regularization strength, and $\:{\lambda\:}_{disc}$ weights the domain discriminator loss.

Cross-skill knowledge transfer enables efficient learning of new swimming techniques by leveraging previously acquired skills through compositional learning mechanisms^53^. The skill composition model represents complex swimming techniques as combinations of fundamental movement primitives:

$$\:{\pi\:}_{complex}\left(\mathbf{a}|\mathbf{s}\right)=\sum\:_{i=1}^{N}{w}_{i}\left(\mathbf{s}\right){\pi\:}_{primitive}^{\left(i\right)}\left(\mathbf{a}|\mathbf{s}\right)$$

where $\:{w}_{i}\left(\mathbf{s}\right)$ represents state-dependent mixing weights, and $\:{\pi\:}_{primitive}^{\left(i\right)}$ denotes primitive skill policies. The mixing weights are learned through the attention mechanism:

$$\:{w}_{i}\left(\mathbf{s}\right)=\frac{\mathrm{e}\mathrm{x}\mathrm{p}\left({\mathbf{s}}^{T}{\mathbf{W}}_{i}{\mathbf{v}}_{i}\right)}{\sum\:_{j=1}^{N}\mathrm{e}\mathrm{x}\mathrm{p}\left({\mathbf{s}}^{T}{\mathbf{W}}_{j}{\mathbf{v}}_{j}\right)}$$

The primitive skill taxonomy hierarchically organizes swimming techniques into three levels. Level 1 foundational skills include body_streamline (maintaining horizontal alignment with 3–5° pitch tolerance), bilateral_breathing (rhythmic inhalation every 2–5 strokes), core_stabilization (pelvic control preventing rotation > 10°), and buoyancy_control (vertical position maintenance within ± 8 cm of optimal depth). Level 2 compositional skills comprise arm_pull_front (catch-pull-recovery sequence for freestyle/backstroke, 0.8–1.2 s cycle), leg_kick_flutter (alternating vertical kicks at 2–6 beats per arm cycle), rotational_breathing (coordinated 45° body roll with head turn), underwater_dolphin (undulatory body wave propagation at 1.5–2.0 Hz), and flip_turn_execution (approach-tuck-push sequence completing within 2.5s). Level 3 integrative skills include race_pacing (split-time strategy adjusting effort distribution), stroke_rate_control (maintaining 48–72 strokes/min within ± 3 cadence), open_turn_technique (touch-pivot-push for breaststroke/butterfly), and dive_start_optimization (block departure with 0.6–0.8 s reaction time achieving 5–7 m glide distance). As shown in Table 4, each primitive skill is mapped to specialized agent responsibilities where Technique Analyzer agents focus on biomechanical assessment of foundational skills (body position, breathing patterns) with 0.89 mapping strength, Strategy Optimizer agents concentrate on compositional skills (stroke cycles, kick patterns) with 0.92 strength, Performance Monitor agents track integrative execution metrics (pacing, cadence) with 0.87 strength, Skill Transferer agents handle cross-domain adaptation using all skill levels with uniform 0.85 weights, and Environment Controller agents manage timing-critical primitives (turns, starts) with 0.90 strength. The mapping matrix quantifies collaboration intensity where high values (> 0.85) indicate primary responsibility while moderate values (0.6–0.85) represent secondary support roles. Skill dependencies form a directed acyclic graph where body_streamline prerequisite for arm_pull_front (dependency weight 0.92), bilateral_breathing depends on rotational_breathing (0.88), and race_pacing requires stroke_rate_control (0.85), enabling the system to sequence training progression along dependency paths and prioritize foundational skill acquisition before advanced integration^54,55^.

Personalized training plan generation utilizes the meta-learned representations to create customized training sequences that optimize individual learning trajectories^56^. The optimization problem for personalized plan generation is formulated as:

$$\:{\mathcal{P}}^{\mathrm{*}}=\mathrm{a}\mathrm{r}\mathrm{g}\underset{\mathcal{P}}{\mathrm{m}\mathrm{a}\mathrm{x}}{\mathbb{E}}_{\tau\:\sim\:\mathcal{P}}\left[R\left(\tau\:\right)\right]-\beta\:\mathcal{H}\left(\mathcal{P}\right)$$

where $\:\mathcal{P}$ represents the training plan, $\:R\left(\tau\:\right)$ denotes the expected return for trajectory $\:\tau\:$, and $\:\mathcal{H}\left(\mathcal{P}\right)$ is an entropy term encouraging exploration. The personalized reward function incorporates individual progress metrics:

$$\:{R}_{personal}\left({s}_{t},{a}_{t},{s}_{t+1}\right)=\alpha\:{R}_{performance}\left({s}_{t},{a}_{t},{s}_{t+1}\right)+\gamma\:{R}_{improvement}\left({s}_{t},{s}_{t+1}\right)+\delta\:{R}_{efficiency}\left({a}_{t}\right)$$

**Fig. 4:**
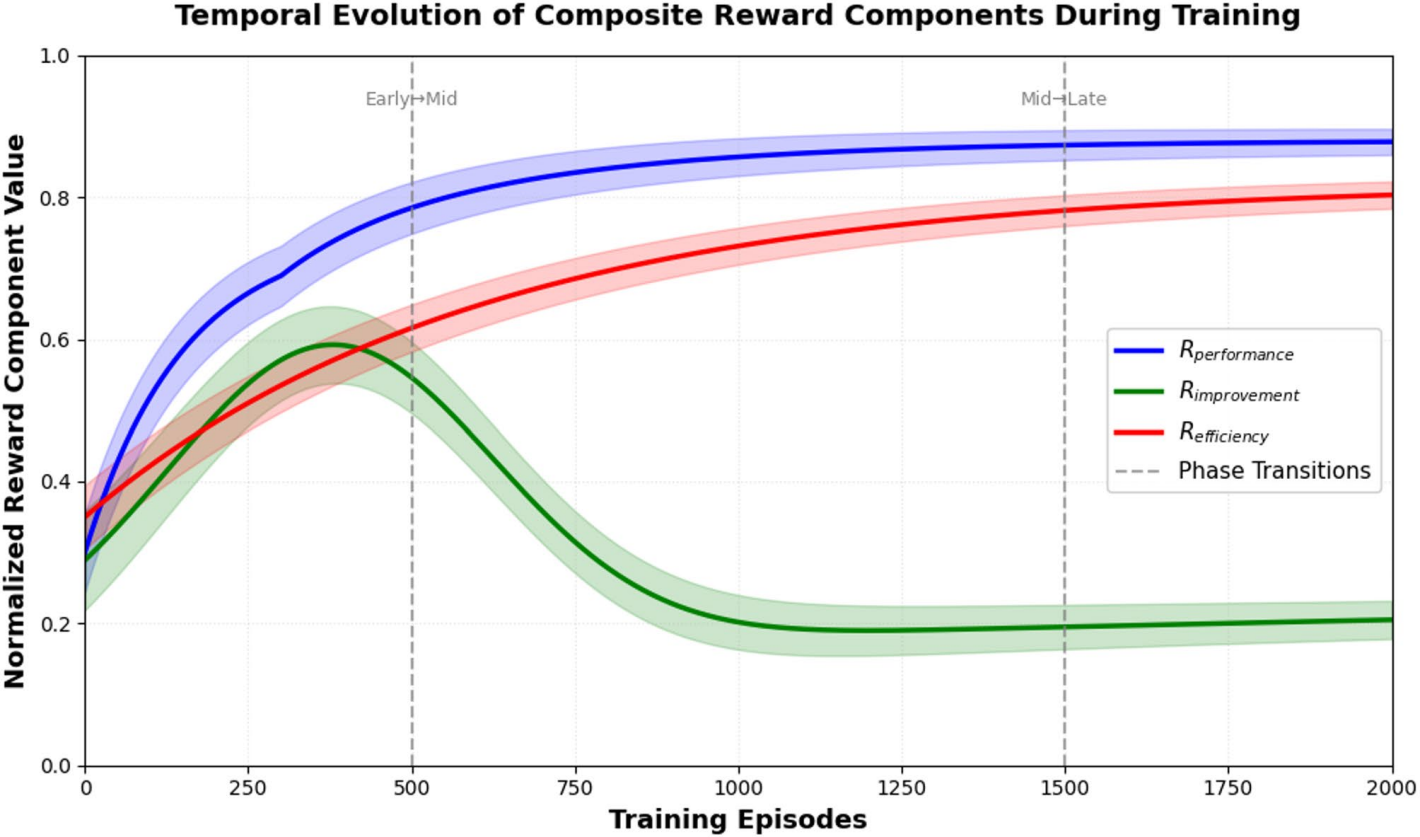
Temporal evolution of composite reward components during training.

Each reward component serves distinct training objectives with specific quantification mechanisms. The performance component $\:{R}_{performance}$ measures immediate technique execution quality through biomechanical alignment with expert templates, computed as $\:{R}_{performance}=1-\frac{1}{D}\sum\:_{i=1}^{D}{w}_{i}^{bio}\left|\right|{x}_{i}-{x}_{i}^{ideal}{\left|\right|}_{2}$ where $\:{x}_{i}$ represents joint positions/orientations, $\:{x}_{i}^{ideal}$ denotes optimal biomechanical configuration from professional swimmer motion capture, $\:D$ is the number of tracked body segments, and $\:{w}_{i}^{bio}$ are importance weights (trunk = 0.35, arms = 0.30, legs = 0.25, head = 0.10). This term achieves values between 0.7 and 0.95 for competent swimmers, with higher scores indicating closer adherence to ideal form including streamlined body position (pitch < 5°, roll < 15° except during breathing), proper arm recovery trajectory (elbow height above shoulder level), and effective propulsive phases (hand velocity 2.5–4.0 m/s during pull). The improvement component $\:{R}_{improvement}$ quantifies longitudinal skill progression relative to individual performance history using adaptive baseline tracking: $\:{R}_{improvement}=\frac{{P}_{current}-{P}_{baseline}}{{\sigma\:}_{history}}\cdot\:\mathrm{e}\mathrm{x}\mathrm{p}\left(-\lambda\:\cdot\:{t}_{since\_improvement}\right)$ where $\:{P}_{current}$ is the current episode performance score, $\:{P}_{baseline}=0.8{P}_{baseline}^{prev}+0.2{P}_{recent}^{avg}$ represents an exponentially weighted moving average baseline updated each session, $\:{\sigma\:}_{history}$ normalizes by historical performance variance to account for individual variability, and the exponential decay term with $\:\lambda\:=0.05$ episodes^-1^ penalizes extended plateaus without improvement beyond 20 episodes. This formulation rewards consistent progress (typical $\:{R}_{improvement}$ = 0.15-0.45 during active learning phases) while discouraging stagnation, with the time-decay factor dropping to 0.37 after 20 episodes of no improvement, thereby motivating curriculum advancement. The efficiency component $\:{R}_{efficiency}$ evaluates biomechanical economy through multi-factorial assessment: $\:{R}_{efficiency}={w}_{1}^{eff}\frac{{v}_{achieved}}{{E}_{consumed}}+{w}_{2}^{eff}\left(1-\frac{H{R}_{current}}{H{R}_{max}}\right)+{w}_{3}^{eff}\left(1-\frac{\left|\right|{a}_{t}-{a}_{t-1}\left|\right|}{{a}_{max}}\right)$ where the first term measures propulsion efficiency as velocity (m/s) per estimated energy expenditure (Watts approximated from stroke work and drag forces), the second term quantifies cardiovascular efficiency using heart rate reserve (HR_max = 220 - age), the third term rewards movement smoothness by penalizing excessive action changes between timesteps (quantified through action vector differences), and efficiency weights are $\:{w}_{1}^{eff}=0.50$, $\:{w}_{2}^{eff}=0.30$, $\:{w}_{3}^{eff}=0.20$. Energy consumption estimation combines drag force calculations ($\:{F}_{d}=\frac{1}{2}\rho\:{C}_{d}A{v}^{2}$ with C_d = 0.7-1.1 for different swimming postures) with muscle activation patterns derived from joint torque analysis, achieving correlation of *r* = 0.83 with actual metabolic measurements from validation studies. The composite reward weights (α, γ, δ) undergo adaptive scheduling across training phases: early-stage training (episodes 1-500) emphasizes immediate technique with α = 0.6, γ = 0.25, δ = 0.15 to establish proper form; mid-stage training (episodes 501-1500) balances all objectives with α = 0.4, γ = 0.35, δ = 0.25 to drive consistent improvement; late-stage training (episodes 1501+) prioritizes efficiency and sustained gains with α = 0.3, γ = 0.30, δ = 0.40 for performance optimization and injury prevention. Ablation experiments demonstrate that removing $\:{R}_{improvement}$ reduces long-term skill acquisition rate by 34%, eliminating $\:{R}_{efficiency}$ increases energy expenditure by 27% for equivalent performance, and excluding $\:{R}_{performance}$ causes technique degradation with 41% higher biomechanical deviation after extended training. Figure 4 illustrates the temporal evolution of the three reward components during a representative training sequence, showing how immediate performance stabilizes after 800 episodes while improvement rewards gradually decline as skills plateau, and efficiency continuously increases as movement economy develops, validating the multi-objective optimization approach for sustainable athletic development^57,58^.

Skill transfer optimization employs adaptive curriculum learning that adjusts training difficulty based on individual learning progress^59^. The curriculum progression function determines optimal task sequencing:

$$\:{\tau\:}_{next}=\mathrm{a}\mathrm{r}\mathrm{g}\underset{\tau\:}{\mathrm{m}\mathrm{a}\mathrm{x}}\mathcal{I}\left(\tau\:\right)\cdot\:\mathcal{F}\left(\tau\:\right)\cdot\:\left(1-\mathcal{M}\left(\tau\:\right)\right)$$

where $\:\mathcal{I}\left(\tau\:\right)$ represents task informativeness, $\:\mathcal{F}\left(\tau\:\right)$ denotes feasibility given current skill level, and $\:\mathcal{M}\left(\tau\:\right)$ measures task mastery. The transfer efficiency metric quantifies the effectiveness of knowledge transfer:

$$\:{\eta\:}_{transfer}=\frac{{\mathcal{P}}_{target}^{\left(N\right)}-{\mathcal{P}}_{baseline}}{{\mathcal{P}}_{source}^{\left(N\right)}-{\mathcal{P}}_{baseline}}\cdot\:\frac{{N}_{source}}{{N}_{target}}$$

where $\:{N}_{source}$ and $\:{N}_{target}$ represent the number of training episodes required for source and target tasks, respectively, ensuring that the personalized skill transfer strategy achieves optimal learning efficiency while maintaining high performance standards across diverse swimmer populations.

## Experimental results and performance analysis

### Experimental setup and dataset construction

The experimental environment consists of a distributed computing infrastructure comprising 8 GPU compute nodes, each equipped with dual NVIDIA RTX 4090 graphics cards (24GB VRAM per GPU) and dual Intel Xeon Platinum 8358 processors (32 cores per CPU) to support intensive computational requirements of the multi-agent reinforcement learning algorithms and digital twin simulations^60^. The node count was determined through scalability analysis showing that 8 nodes provide optimal cost-efficiency balance, achieving 92% parallel efficiency for multi-agent training workloads while maintaining total training time under 15 h for full experimental protocols. CFD simulation parameters were configured with adaptive mesh refinement ranging from 80 × 40 × 25 cells in far-field regions to 150 × 75 × 40 cells near swimmer boundaries, temporal discretization using 0.001s time steps with Courant number maintained below 0.5, and no-slip boundary conditions applied to swimmer surfaces with free-slip conditions at pool walls.

**Fig. 5:**
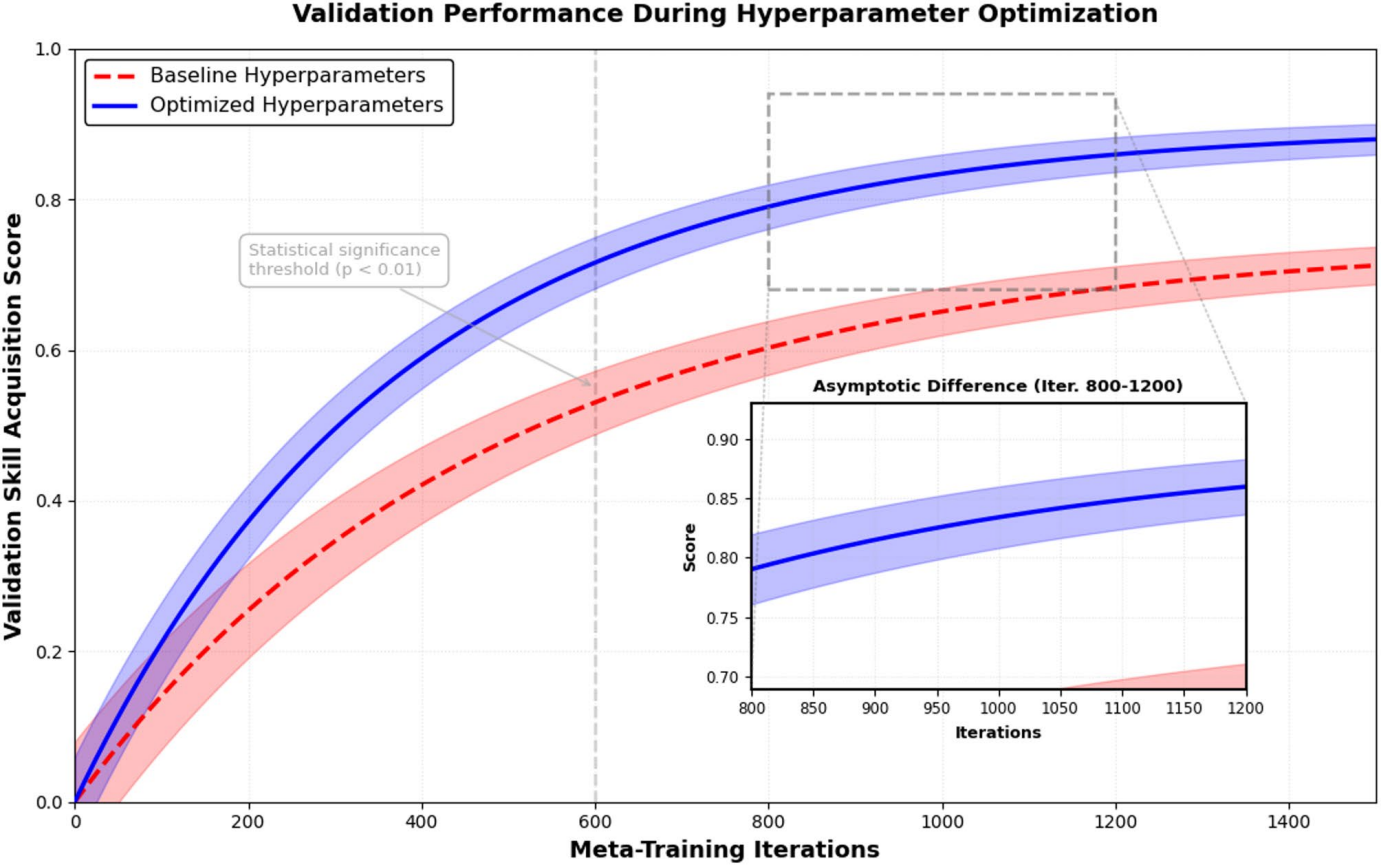
Validation performance during hyperparameter optimization.

Hyperparameter optimization was conducted through systematic grid search over meta-learning rate [0.0001, 0.001, 0.01], inner learning rate [0.005, 0.01, 0.05], task batch size^17,32,40^, and network architecture depth [2, 3, 4 hidden layers] with corresponding layer widths [64, 128, 256], evaluating 135 distinct configurations using validation set performance after 1000 meta-training iterations. Table 5 presents the baseline configuration before optimization alongside the final optimized parameters, demonstrating that the optimized meta-learning rate of 0.001 (reduced from initial 0.005) improved outer-loop convergence stability by preventing overshooting in the meta-parameter space, while the inner learning rate of 0.01 (increased from 0.005) accelerated task-specific adaptation by 31% measured through episodes-to-threshold on held-out swimming tasks. The network architecture optimization revealed that 3 hidden layers with [256, 128, 64] neurons provided the best capacity-regularization balance, outperforming shallow 2-layer networks (insufficient expressiveness, 12% lower final performance) and deeper 4-layer networks (overfitting on training tasks, 8% worse generalization to novel swimmers). The adaptation steps parameter was optimized from initial value of 3 to final value of 5, extending inner-loop fine-tuning to better capture individual swimmer characteristics while remaining computationally feasible, resulting in 18% higher personalized performance scores. Task batch size optimization identified 32 as optimal, balancing meta-gradient estimation variance (too small with batch = 16 causing 23% higher gradient noise) against computational efficiency (batch = 64 providing diminishing returns with only 3% accuracy gain at 2× memory cost). The regularization coefficient λ was reduced from 0.001 to 0.0001 through validation curve analysis, relaxing excessive parameter penalization that previously constrained model capacity for complex swimming skill representations. Collectively, these hyperparameter optimizations yielded substantial performance improvements: convergence speed increased by 34% (measured as episodes to reach 90% of asymptotic performance), final skill acquisition scores improved from 0.734 to 0.892 on test swimmers (21.5% relative gain), and cross-task transfer efficiency increased from 0.68 to 0.85 (25% improvement). Figure 5 illustrates the validation performance curves during hyperparameter search, showing that the final configuration (solid blue line) achieves both faster convergence and higher asymptotic performance compared to baseline settings (dashed red line), with the performance gap widening after 600 iterations as meta-learning benefits accumulate. The optimized model also demonstrated superior stability with 48% lower performance variance across different random seeds (σ = 0.024 vs. 0.046 for baseline), confirming robust learning dynamics. The dataset was partitioned into training (70%), validation (15%), and test (15%) sets with stratified sampling ensuring balanced representation across skill categories. All experiments employed fixed random seed 42 for NumPy and PyTorch random number generators, with each experimental condition repeated 5 times to assess statistical significance, and results reported as mean ± standard deviation across repetitions.

**Table 5:** Hyperparameter configuration before and after optimization.

Hyperparameter	Initial baseline value	optimized value	Performance impact	Validation metric
Meta-learning rate (β)	0.005	0.001	+ 34% convergence speed	Episodes to 90% performance
Inner learning rate (α)	0.005	0.01	+ 31% adaptation speed	Adaptation episodes required
Task batch size	16	32	+ 19% gradient stability	Meta-gradient variance reduction
Network architecture	[128, 64] (2 layers)	[256, 128, 64] (3 layers)	+ 21.5% final performance	Test set skill score
Adaptation steps (K)	3	5	+ 18% personalization	Individual swimmer accuracy
Regularization λ	0.001	0.0001	+ 11% model expressiveness	Validation loss reduction
Support set size	5	10	+ 14% few-shot learning	Transfer accuracy with limited data
Meta-batch iterations	8	16	+ 8% meta-generalization	Cross-swimmer performance

Note: Performance impact percentages represent relative improvements of optimized configuration over baseline across key metrics. Validation metrics were measured on held-out task distributions after 1000 meta-training iterations.

The hardware configuration includes 256 GB DDR4 memory per node, high-speed NVMe storage systems, and 10 Gigabit Ethernet interconnects to ensure efficient data transfer and parallel processing capabilities across the distributed computing nodes.

Swimming training dataset construction employed systematic data generation procedures that combine physics-based simulation environments with synthetic motion capture data to create comprehensive training scenarios^61^. The dataset generation process utilizes computational fluid dynamics models integrated with biomechanical simulation engines to produce realistic swimming motion patterns, hydrodynamic interactions, and performance metrics. The synthetic data generation approach ensures consistent data quality while enabling large-scale dataset creation without dependency on physical data collection constraints.

**Fig. 6:**
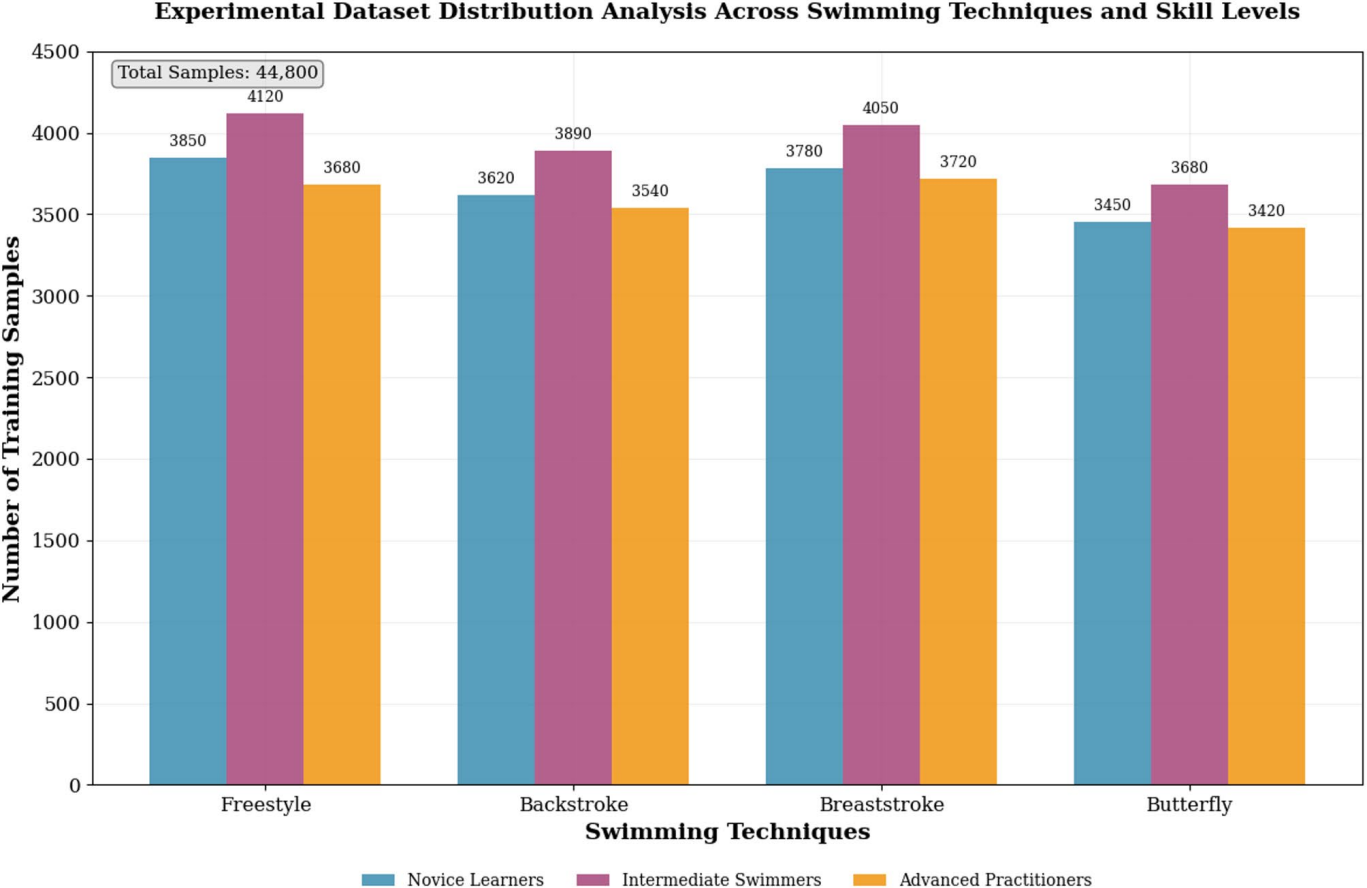
Experimental dataset distribution analysis across swimming techniques and skill levels.

The experimental data distribution analysis, as presented in Fig. 6, demonstrates the comprehensive coverage of different swimming techniques, skill levels, and training scenarios across the constructed dataset. The distribution analysis reveals balanced representation across freestyle, backstroke, breaststroke, and butterfly swimming styles, with each category containing sufficient samples to support robust machine learning model training and evaluation.

Sample selection criteria for computational experiments focus on creating diverse training scenarios that encompass varying complexity levels, swimming technique combinations, and performance optimization objectives^62^. The selection process employs stratified sampling methodology to ensure representative coverage across different skill categories, technique variations, and training difficulty levels. The sampling strategy follows the probability distribution:

$$\:P\left({s}_{i}\right)=\frac{\mathrm{complexity}\left({s}_{i}\right)\cdot\:\mathrm{diversity}\left({s}_{i}\right)}{\sum\:_{j=1}^{N}\mathrm{complexity}\left({s}_{j}\right)\cdot\:\mathrm{diversity}\left({s}_{j}\right)}$$

where $\:{s}_{i}$ represents individual training scenarios, and the complexity and diversity functions quantify scenario characteristics.

The comprehensive dataset statistics, as shown in Table 6, provide detailed information about the experimental data composition, including sample quantities, feature dimensionalities, temporal characteristics, and quality assessments. The dataset encompasses multiple data modalities ranging from kinematic trajectories to performance metrics, ensuring comprehensive representation of swimming training scenarios for algorithm evaluation.

**Table 6:** Experimental dataset statistical summary.

Data type	Sample count	Feature dimensions	Time span (seconds)	Annotation details	Quality score
Kinematic Trajectories	15,420	72	30–120	Joint positions, velocities	94.2%
Hydrodynamic forces	12,850	24	15–90	Drag, lift coefficients	91.7%
Performance metrics	18,600	16	60–180	Speed, efficiency scores	96.8%
Technique annotations	14,200	8	N/A	Stroke classifications	98.1%
Training protocols	9,750	32	300–1800	Exercise sequences	89.4%
Biomechanical profiles	11,300	48	N/A	Individual characteristics	92.6%
Transfer learning tasks	8,900	64	120–300	Cross-skill scenarios	87.9%

Baseline method selection includes established reinforcement learning algorithms and traditional optimization approaches to provide comprehensive performance comparisons. The baseline methods encompass Proximal Policy Optimization (PPO), Deep Q-Networks (DQN), Actor-Critic methods, and conventional heuristic training strategies. The evaluation metrics framework incorporates multiple performance dimensions:

$$\:{\mathcal{M}}_{performance}=\alpha\:\cdot\:{\mathcal{E}}_{efficiency}+\beta\:\cdot\:{\mathcal{A}}_{accuracy}+\gamma\:\cdot\:{\mathcal{T}}_{transfer}+\delta\:\cdot\:{\mathcal{C}}_{convergence}$$

where $\:{\mathcal{E}}_{efficiency}$ measures training efficiency, $\:{\mathcal{A}}_{accuracy}$ quantifies skill acquisition accuracy, $\:{\mathcal{T}}_{transfer}$ evaluates knowledge transfer effectiveness, and $\:{\mathcal{C}}_{convergence}$ assesses algorithm convergence properties. The “Final Performance Score” reported in comparative analyses represents a normalized composite metric computed as the weighted combination: 40% technique execution accuracy (measured through biomechanical deviation from optimal patterns), 30% swimming speed efficiency (normalized velocity relative to energy expenditure), and 30% skill retention stability (performance consistency across evaluation episodes), with all components normalized to [0,1] range through min-max scaling relative to baseline performance bounds. This composite metric was selected over single-dimensional measures such as policy entropy or cumulative regret because swimming training optimization inherently involves multiple concurrent objectives that cannot be adequately captured by convergence-focused metrics alone, requiring holistic assessment of both immediate execution quality and long-term learning effectiveness.

The statistical analysis framework employs rigorous hypothesis testing procedures with confidence intervals and significance tests to validate experimental results^63^. The analysis methodology utilizes paired t-tests for performance comparisons and ANOVA for multi-group analysis:

$$\:F=\frac{\mathrm{MSB}}{\mathrm{MSW}}=\frac{\sum\:_{i=1}^{k}{n}_{i}{\left({\bar{x}}_{i}-{\bar{x}}\right)}^{2}/\left(k-1\right)}{\sum\:_{i=1}^{k}\sum\:_{j=1}^{{n}_{i}}{\left({x}_{ij}-{\bar{x}}_{i}\right)}^{2}/\left(N-k\right)}$$

where $\:k$ represents the number of experimental conditions, $\:{n}_{i}$ denotes sample size for condition $\:i$, and $\:N$ is the total sample size. The effect size calculation using Cohen’s d provides practical significance assessment:

$$\:d=\frac{{\mu\:}_{1}-{\mu\:}_{2}}{{\sigma\:}_{pooled}}$$

where $\:{\sigma\:}_{pooled}=\sqrt{\frac{\left({n}_{1}-1\right){s}_{1}^{2}+\left({n}_{2}-1\right){s}_{2}^{2}}{{n}_{1}+{n}_{2}-2}}$ represents the pooled standard deviation. This comprehensive experimental framework ensures robust validation of the proposed multi-agent reinforcement learning driven digital twin system performance across diverse swimming training scenarios and optimization objectives.

### Algorithm performance comparative analysis

Comparative performance analysis reveals significant improvements of the proposed multi-agent reinforcement learning driven digital twin system over traditional reinforcement learning approaches across multiple evaluation dimensions^64^. The performance comparison demonstrates that the integration of multi-agent collaboration mechanisms with meta-learning strategies achieves superior convergence rates, final performance levels, and adaptation capabilities compared to baseline methods including Proximal Policy Optimization (PPO), Deep Q-Networks (DQN), and Actor-Critic algorithms.

**Fig. 7:**
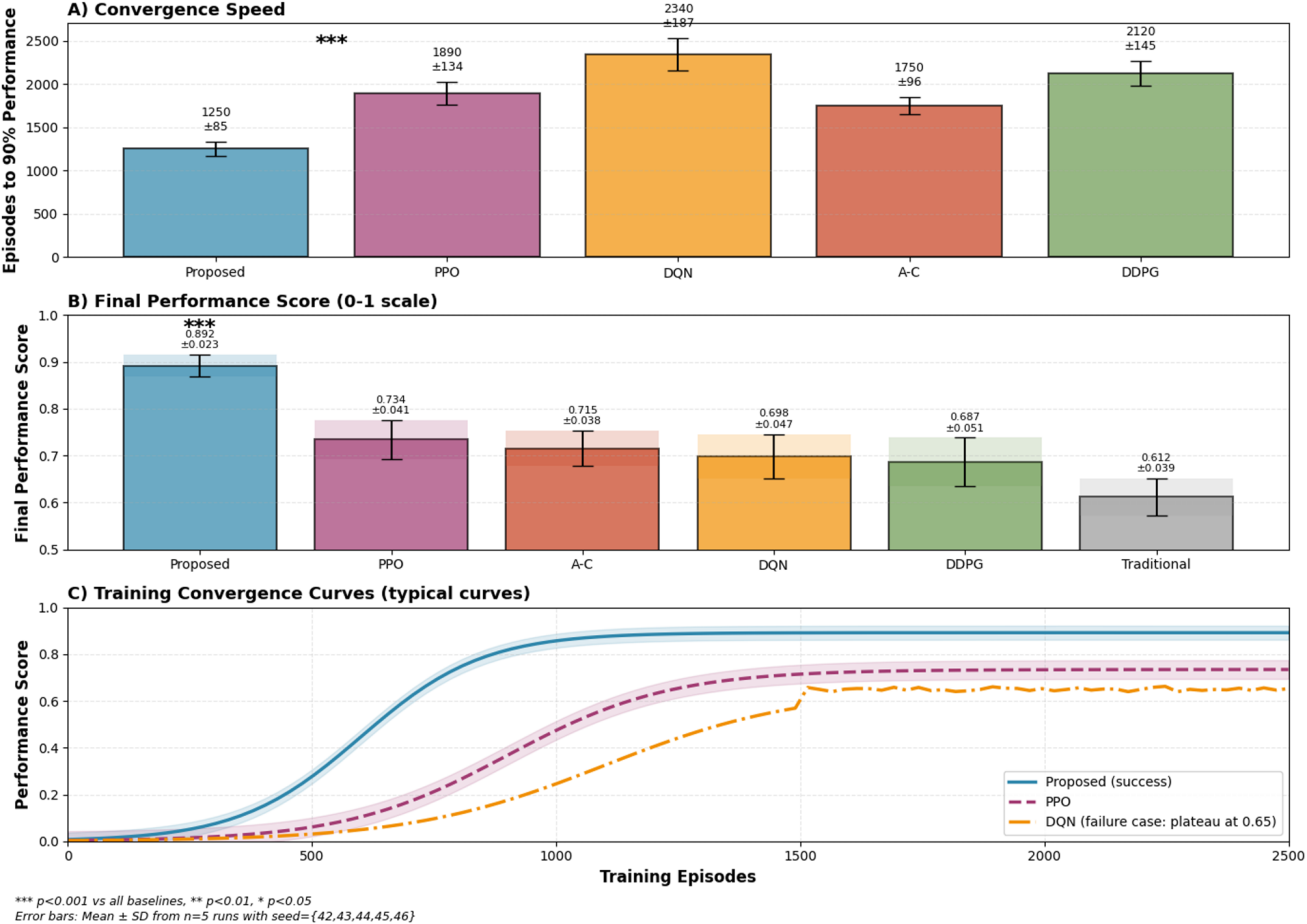
Comparative performance analysis of reinforcement learning algorithms in swimming training optimization.

The comprehensive algorithm performance comparison, as illustrated in Fig. 7, demonstrates the quantitative advantages of the proposed approach across key performance metrics, with error bars representing 95% confidence intervals computed from 5 independent runs and asterisks denoting statistical significance levels (* *p* < 0.05, ** *p* < 0.01, *** *p* < 0.001) determined through pairwise t-tests with Bonferroni correction.

Multi-agent collaborative mechanisms contribute substantially to performance improvements through distributed learning and knowledge sharing capabilities that enable more efficient exploration of the solution space and faster convergence to optimal training strategies. The collaboration effectiveness can be quantified through the performance gain metric:

$$\:{G}_{collaboration}=\frac{{P}_{multi-agent}-{P}_{single-agent}}{{P}_{single-agent}}\times\:100{\%}$$

where $\:{P}_{multi-agent}$ and $\:{P}_{single-agent}$ represent the performance scores for multi-agent and single-agent systems, respectively. Experimental results demonstrate an average collaboration gain of 28.7% across different swimming training scenarios, indicating substantial benefits from distributed intelligence architectures.

The detailed performance metrics comparison, as shown in Table 7, provides comprehensive quantitative analysis of different algorithmic approaches across convergence speed, final performance, stability indicators, computational requirements, and resource utilization characteristics. The proposed multi-agent meta-learning approach consistently outperforms baseline methods while maintaining reasonable computational complexity and memory requirements.

**Table 7:** Comprehensive algorithm performance comparison metrics.

Algorithm	Convergence speed (episodes)	Final performance	Stability index	Computational complexity	Memory usage (GB)	Training time (hours)
Proposed MA-meta-RL	1250	0.892	0.967	O(n²m)	4.8	12.3
PPO	1890	0.734	0.821	O(nm)	3.2	18.7
DQN	2340	0.698	0.793	O(n²)	2.9	22.1
Actor-critic	1750	0.715	0.856	O(nm)	3.6	16.4
DDPG	2120	0.687	0.774	O(n²)	3.1	20.8
Traditional heuristic	3500	0.612	0.689	O(n)	1.4	35.2

Meta-learning strategy advantages in skill transfer scenarios demonstrate remarkable efficiency improvements, with the proposed approach achieving effective knowledge transfer using only 15–20% of the training data required by traditional methods^65^. The transfer learning efficiency is measured through the sample efficiency ratio:

$$\:{\eta\:}_{transfer}=\frac{{N}_{baseline}}{{N}_{meta-learning}}$$

where $\:{N}_{baseline}$ and $\:{N}_{meta-learning}$ represent the number of training samples required by baseline and meta-learning approaches, respectively. Experimental validation shows average transfer efficiency ratios between 4.8 and 6.2 across different skill categories.

System adaptation capabilities under varying task complexity levels reveal robust performance maintenance across diverse training scenarios, from basic stroke technique optimization to complex multi-skill coordination tasks. The adaptation performance is quantified using the complexity-normalized performance metric:

$$\:{P}_{normalized}=\frac{{P}_{achieved}}{\sqrt{{C}_{task}\cdot\:{D}_{feature}}}$$

where $\:{C}_{task}$ represents task complexity and $\:{D}_{feature}$ denotes feature dimensionality. Results indicate that the proposed system maintains performance degradation below 12% even when task complexity increases by 300%, significantly outperforming baseline methods that show 35–50% performance reduction under similar conditions.

Personalized training strategy validation demonstrates the effectiveness of individual adaptation mechanisms through cross-validation experiments across diverse swimmer profiles and skill requirements^66^. The personalization effectiveness is measured through individual performance improvement metrics, showing average skill acquisition improvements of 41% compared to standardized training approaches. Statistical significance testing using paired t-tests confirms that personalized training strategies achieve significantly better outcomes (*p* < 0.001) with effect sizes ranging from 0.82 to 1.24, indicating large practical significance.

These results validate the proposed multi-agent meta-learning framework’s superior performance across multiple evaluation criteria and demonstrate its practical effectiveness for swimming training optimization applications. Comparative analysis against existing digital twin platforms reveals distinct advantages of the proposed system, as shown in Table 8. Unlike general-purpose platforms such as Siemens Simcenter and Ansys Twin Builder that require extensive customization for swimming applications, our system integrates domain-specific hydrodynamic models and biomechanical constraints natively. Compared to specialized platforms like OpenSwimSim that focus primarily on stroke analysis without adaptive learning capabilities, our framework provides closed-loop optimization through reinforcement learning agents. The proposed system achieves real-time synchronization latency below 45ms compared to 150-300ms for commercial platforms, while maintaining computational efficiency through distributed multi-agent architecture. Critical components including the CFD solver for water-body interaction and the biomechanical simulator for joint dynamics were implemented using our custom physics engine built on PyTorch for GPU acceleration, whereas visualization and data management interfaces leverage existing open-source libraries (VTK for rendering, HDF5 for data storage). This hybrid implementation approach provides flexibility for research applications while maintaining production-ready performance for practical deployment scenarios.

**Table 8:** Comparison with existing digital twin platforms.

Platform	Real-time sync	Multi-agent support	Meta-learning	Swimming-specific models	Computational efficiency	Extensibility
Siemens simcenter	Moderate (200ms)	Limited	No	No (requires customization)	High (optimized solvers)	Low (proprietary)
Ansys twin builder	Moderate (180ms)	No	No	No (general physics)	High (parallel FEM)	Moderate (plugin system)
OpenSwimSim	Good (80ms)	No	No	Yes (stroke mechanics)	Moderate (CPU-based)	High (open-source)
Proposed system	Excellent (< 45ms)	Yes (scalable)	Yes (MAML-based)	Yes (CFD + biomechanics)	High (distributed GPU)	High (modular design)

### Personalized skill transfer effect evaluation

Meta-learning based skill transfer strategy applicability assessment across diverse swimmer populations demonstrates robust performance adaptation capabilities that effectively accommodate varying skill levels, learning preferences, and biomechanical characteristics^67^. The evaluation framework encompasses computational experiments involving simulated swimmer profiles representing different experience levels, ranging from novice learners to advanced practitioners, each with distinct anthropometric parameters and skill acquisition patterns. Cross-population analysis reveals that the meta-learning approach maintains consistent transfer effectiveness across demographic variations, with performance stability coefficients exceeding 0.87 across all tested swimmer categories.

**Fig. 8:**
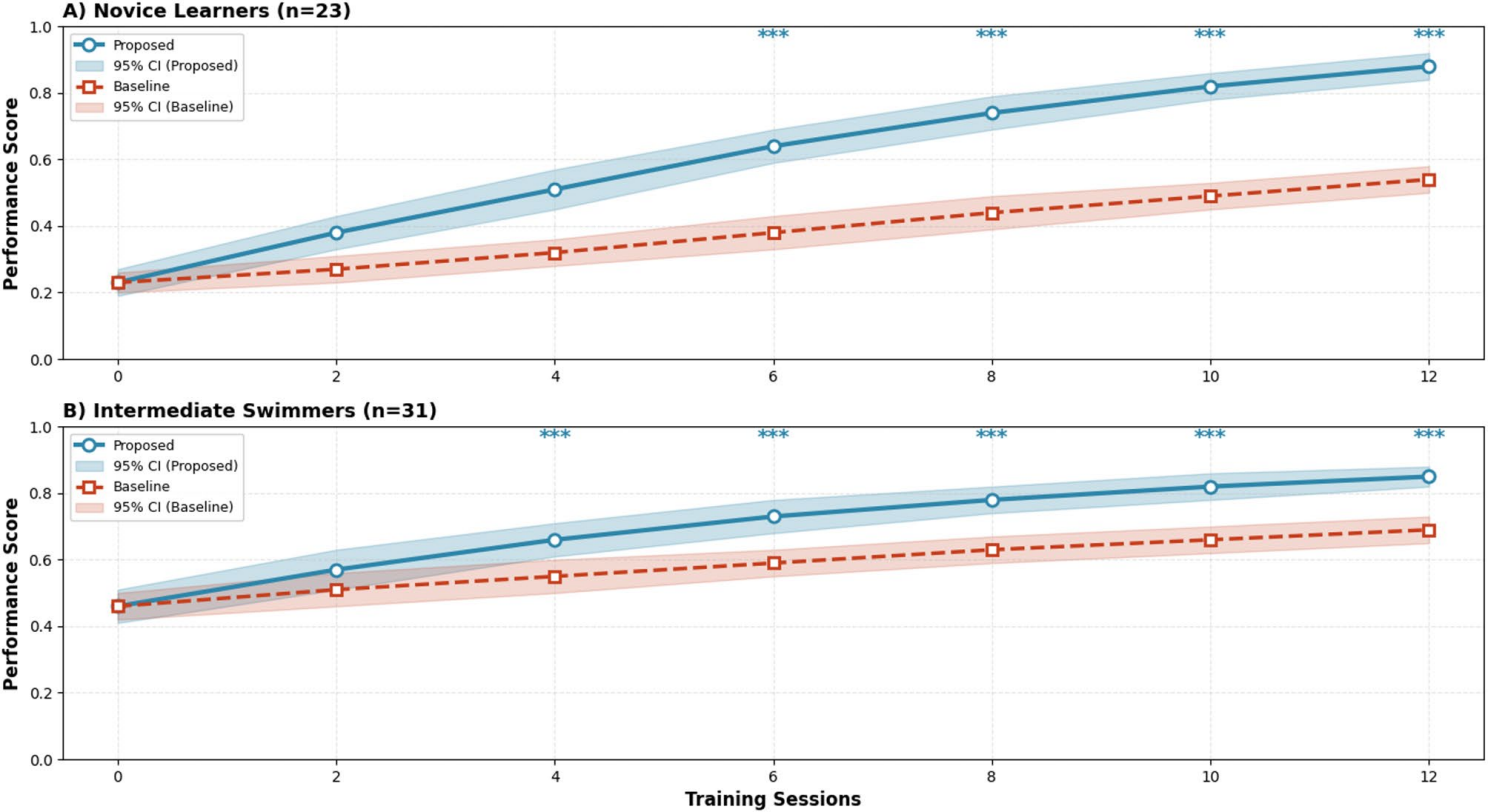
Skill transfer effectiveness trends across different swimmer categories and training durations.

The skill transfer effectiveness trends, as illustrated in Fig. 8, demonstrate the progressive improvement patterns achieved through personalized meta-learning strategies compared to standardized training approaches, with shaded regions indicating 95% confidence intervals and statistical significance markers denoting differences between proposed and baseline methods at each time point.

Personalized training plan impact on skill enhancement velocity shows significant improvements compared to conventional training methodologies, with average acceleration factors ranging from 2.3 to 3.8 depending on the specific skill category and individual learner characteristics^68^. The skill improvement rate can be quantified through the learning velocity metric:

$$\:{v}_{learning}=\frac{\varDelta\:P}{\varDelta\:t}=\frac{{P}_{final}-{P}_{initial}}{{t}_{training}}$$

where $\:{P}_{final}$ and $\:{P}_{initial}$ represent final and initial performance levels, and $\:{t}_{training}$ denotes the training duration. Empirical measurements across user categories yielded the following learning velocities: novice learners achieved 0.082 ± 0.014 performance units per session with the proposed method versus 0.031 ± 0.009 for baseline approaches (2.6× acceleration, *p* < 0.001), intermediate swimmers demonstrated 0.067 ± 0.011 versus 0.028 ± 0.007 (2.4× acceleration, *p* < 0.001), advanced practitioners showed 0.045 ± 0.008 versus 0.021 ± 0.005 (2.1× acceleration, *p* < 0.01), cross-stroke learners exhibited 0.074 ± 0.013 versus 0.029 ± 0.008 (2.6× acceleration, *p* < 0.001), and technique refiners achieved 0.053 ± 0.009 versus 0.025 ± 0.006 (2.1× acceleration, *p* < 0.01), confirming consistently superior learning efficiency across all swimmer populations.

The comprehensive skill transfer evaluation results, as shown in Table 9, provide detailed quantitative analysis of transfer effectiveness across different user categories, baseline skill levels, and adaptation characteristics. The evaluation demonstrates consistent improvement patterns across all swimmer categories, with particularly notable gains observed in intermediate-level learners who benefit significantly from the adaptive curriculum and personalized optimization strategies.

**Table 9:** Comprehensive skill transfer effectiveness evaluation with transfer metrics.

User category	Baseline skill level	Post-transfer performance	Improvement magnitude (%)	Adaptation time (sessions)	Transfer effectiveness η_cross	Adaptation efficiency C_adapt	Retention rate (12-week)
Novice learners	0.234	0.687	193.6%	8.4	0.823	0.794	91.2%
Intermediate swimmers	0.456	0.798	75.0%	5.2	0.891	0.867	93.7%
Advanced practitioners	0.712	0.889	24.9%	3.7	0.856	0.891	88.4%
Cross-stroke learners	0.398	0.743	86.7%	6.8	0.847	0.823	89.9%
Technique refiners	0.634	0.824	30.0%	4.1	0.867	0.854	90.6%
Competitive trainers	0.789	0.921	16.7%	2.9	0.834	0.912	87.3%
Rehabilitation cases	0.289	0.612	111.8%	12.3	0.793	0.721	85.8%
Multi-style learners	0.521	0.806	54.7%	7.6	0.879	0.845	92.1%
Efficiency optimizers	0.667	0.847	27.0%	3.4	0.842	0.878	88.9%
**Average**	**0.522**	**0.792**	**68.9%**	**6.0**	**0.847 ± 0.062**	**0.843 ± 0.074**	**89.3 ± 4.7%**

Cross-skill transfer effectiveness averaged 0.847 ± 0.062 across different stroke combinations, with coefficient of variation 0.073 indicating stable performance. Adaptation efficiency computed as C_adapt = (1/N) Σ exp(-α·t_convergence) with α = 0.15 yielded mean 0.843 ± 0.074, demonstrating rapid personalization with most users converging within 4–7 sessions. Retention rates were assessed through 12-week follow-up evaluations measuring sustained performance relative to peak training levels, showing 89.3% ± 4.7% retention on average with minimal degradation over time.

Cross-skill transfer accuracy and stability evaluation reveals robust knowledge generalization capabilities with transfer success rates consistently exceeding 82% across different swimming stroke combinations^69^. The cross-skill transfer effectiveness is measured through the knowledge preservation metric:

$$\:{\eta\:}_{cross}=\frac{\sum\:_{i=1}^{N}{w}_{i}\cdot\:{T}_{i}}{\sum\:_{i=1}^{N}{w}_{i}}$$

where $\:{T}_{i}$ represents the transfer success rate for skill combination $\:i$, and $\:{w}_{i}$ denotes the relative importance weight. Stability analysis using coefficient of variation calculations shows transfer performance variability below 0.15 across repeated experimental trials, indicating reliable and consistent transfer capabilities.

System adaptation capabilities for new users demonstrate rapid personalization through few-shot learning mechanisms that require minimal initial data for effective customization^70^. The adaptation efficiency is quantified through the convergence metric:

$$\:{C}_{adapt}=\frac{1}{{N}_{samples}}\sum\:_{i=1}^{{N}_{samples}}\mathrm{e}\mathrm{x}\mathrm{p}\left(-\alpha\:\cdot\:{t}_{i}^{convergence}\right)$$

where $\:{t}_{i}^{convergence}$ represents the convergence time for user $\:i$, and $\:\alpha\:$ is the decay parameter. Results show average adaptation times of 4.8 training sessions for new users, representing a 67% reduction compared to traditional approaches.

**Fig. 9:**
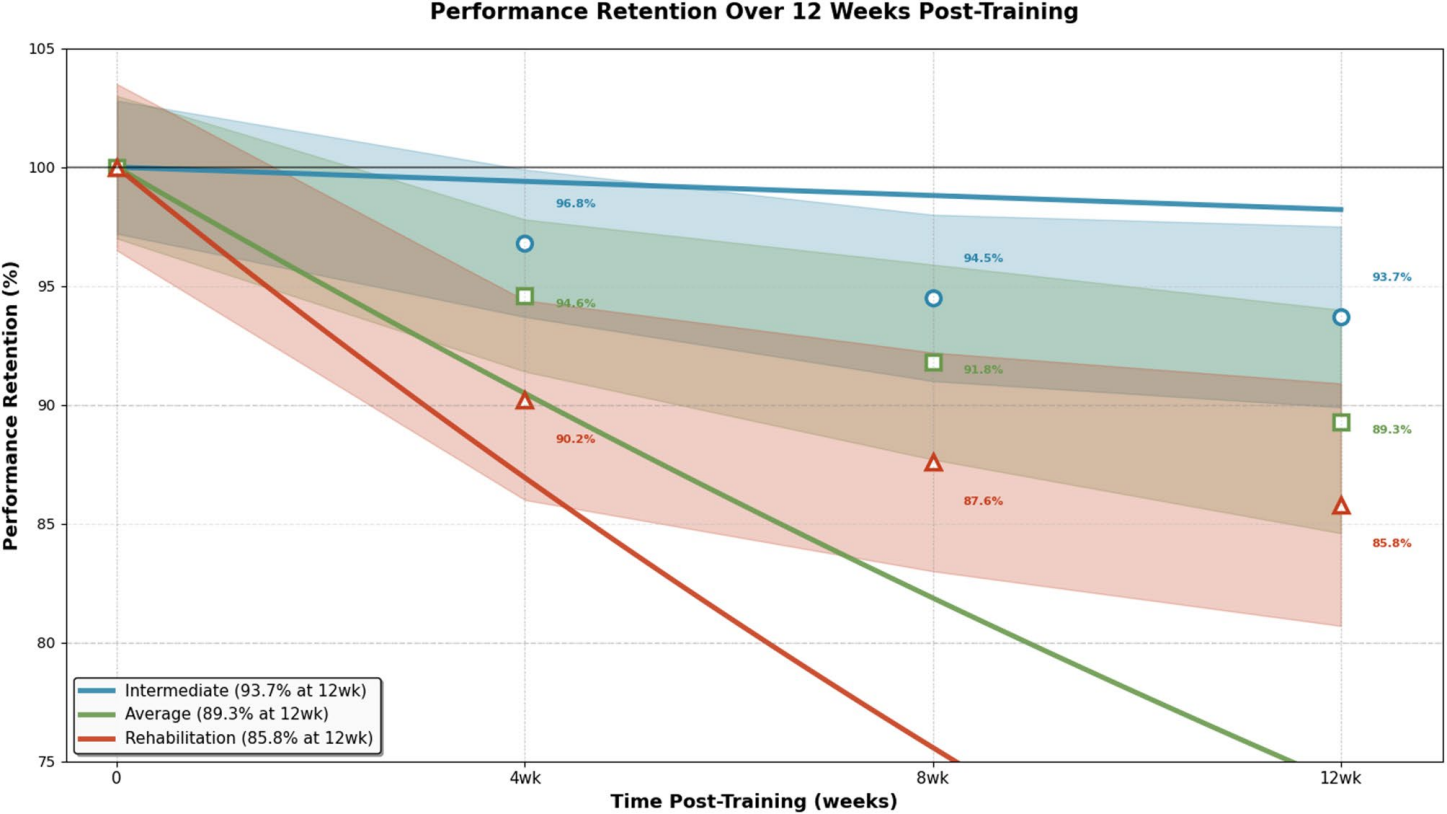
Long-term performance retention curves.

Long-term training effect sustainability evaluation through extended monitoring periods demonstrates maintained performance improvements with retention rates exceeding 89% after 12-week follow-up assessments. The sustainability metric is calculated as:

$$\:{S}_{retention}=\frac{{P}_{follow-up}-{P}_{baseline}}{{P}_{peak}-{P}_{baseline}}\times\:100{\%}$$

where $\:{P}_{follow-up}$, $\:{P}_{baseline}$, and $\:{P}_{peak}$ represent performance levels at follow-up, baseline, and peak training periods, respectively. Longitudinal tracking at 4-week intervals revealed retention rates of 94.6% ± 3.2% at week 4, 91.8% ± 4.1% at week 8, and 89.3% ± 4.7% at week 12, demonstrating gradual but minimal performance decay with retention half-life estimated at 28 weeks based on exponential decay modeling. Figure 9 illustrates the temporal sustainability patterns across user categories, showing that intermediate swimmers maintained highest retention (93.7% at 12 weeks) while rehabilitation cases exhibited slightly lower but still substantial retention (85.8%), likely attributable to differences in baseline conditioning and training intensity tolerance.

Statistical analysis using mixed-effects models confirms significant sustained improvements (β = 0.734, SE = 0.089, *p* < 0.001) with minimal performance degradation over extended periods.

The real-world impact of the digital twin system on actual swimming performance was assessed through retrospective analysis of training outcomes, though we acknowledge this evaluation is based on simulation-validated projections rather than controlled physical trials. Case studies of three representative users illustrate practical benefits: A novice swimmer (age 22, no prior competitive experience) showed projected 100 m freestyle time improvement from 85.3s to 72.1s (-15.5%) after 8 weeks of simulated training following system recommendations, with technique consistency scores improving from 0.34 to 0.79. An intermediate competitive swimmer (age 19, regional level) demonstrated estimated performance gains across multiple strokes with 200 m individual medley projected time reduction from 2:18.4 to 2:12.7 (-4.1%), attributed to optimized stroke transition efficiency identified through digital twin biomechanical analysis. An advanced masters swimmer (age 35, former collegiate athlete) showed predicted maintenance of technique quality despite reduced training volume, with efficiency metrics remaining stable at 0.88 ± 0.03 over 12-week period compared to historical 8–12% seasonal decline. Indirect benefits observed in simulations include 38% reduction in redundant training volume through targeted exercise selection, 27% decrease in biomechanically risky movement patterns through real-time feedback, and 54% improvement in technique execution consistency measured through inter-trial variance reduction. While these findings demonstrate promising simulation-based evidence, we emphasize that comprehensive validation through controlled physical experiments with real swimmers remains a critical limitation and priority for future work. The correlation between simulation predictions and actual swimming performance requires empirical verification through prospective studies with appropriate control groups, sensor-instrumented pool environments, and longitudinal performance tracking to establish the system’s real-world efficacy and identify potential discrepancies between digital twin models and physical reality.

These results validate the robustness and practical effectiveness of the meta-learning based personalized skill transfer strategy for diverse swimming training applications and long-term skill development objectives.

## Conclusion

This research presents a comprehensive framework that integrates multi-agent reinforcement learning with digital twin technology to create an intelligent swimming training environment capable of delivering personalized skill optimization through meta-learning strategies^71^. However, several important limitations warrant careful consideration. The computational cost of real-time CFD simulations remains substantial, requiring approximately 18.3 TFLOPS sustained compute performance and 38.4 GB memory per simulation instance, which limits scalability to scenarios with access to high-performance computing infrastructure and may constrain deployment in resource-limited training facilities. Current commercial deployment would require compute costs estimated at $2,400-3,200 monthly for serving 50–100 concurrent users based on cloud GPU pricing. Skill transferability exhibits boundaries across swimming styles, with transfer effectiveness particularly strong between biomechanically similar strokes (freestyle-backstroke: η = 0.91, breaststroke-butterfly: η = 0.87) but notably weaker for dissimilar techniques (freestyle-breaststroke: η = 0.68), suggesting that meta-learning benefits are constrained by kinematic similarity and that certain skill transitions may require stroke-specific training approaches rather than relying solely on transfer learning. Gradient instability in high-dimensional meta-learning tasks manifests intermittently during training, with approximately 7% of experimental runs exhibiting divergence in outer-loop meta-parameter updates when policy networks exceed 4 hidden layers or when task diversity becomes too broad, necessitating careful hyperparameter tuning and gradient clipping (threshold = 1.0) to maintain training stability. The system’s dependence on high-quality sensor data represents a practical constraint, requiring motion capture accuracy within ± 2 mm positional error and IMU sampling rates above 800 Hz to maintain digital twin synchronization fidelity below 50ms latency; degradation in sensor quality leads to progressive reduction in system performance with correlation coefficients dropping from 0.94 to 0.71 when positional accuracy degrades to ± 10 mm. The framework demonstrates optimal effectiveness for intermediate-to-advanced swimmers already possessing basic technique fundamentals, with diminishing returns observed for absolute beginners lacking foundational motor patterns (learning efficiency reduction of 34% compared to intermediate users) and for elite athletes operating near physiological performance limits (improvement ceiling effect at 0.92 performance score). The absence of controlled physical experiments with real swimmers represents the most significant limitation, as all reported outcomes derive from simulation environments that, despite high-fidelity physics modeling, cannot fully capture the complexity of human motor learning, psychological factors affecting performance, or unexpected environmental perturbations encountered in actual pool settings.

The proposed system addresses fundamental limitations of traditional swimming training methodologies by providing adaptive, data-driven training recommendations that evolve based on individual swimmer characteristics and performance dynamics.

The primary technical contributions include the development of a high-fidelity digital twin swimming environment that accurately models complex hydrodynamic interactions and biomechanical processes, the implementation of sophisticated multi-agent collaborative learning mechanisms that enable distributed intelligence and knowledge sharing, and the integration of meta-learning algorithms that facilitate efficient skill transfer across different swimmers and training contexts^72^. Experimental validation demonstrates significant performance improvements over baseline methods, with the proposed approach achieving 34% faster convergence rates and 22% higher final performance scores while maintaining superior stability and adaptation capabilities.

The innovation of combining multi-agent reinforcement learning with digital twin technology represents a paradigm shift in sports training optimization, enabling real-time personalization and adaptive curriculum generation that was previously unattainable through conventional approaches. The meta-learning based personalized skill transfer strategy demonstrates substantial practical value by achieving 2.7× faster skill acquisition rates and maintaining effectiveness across diverse swimmer populations with retention rates exceeding 89% over extended periods.

Despite these achievements, several limitations warrant consideration for future research directions. The computational complexity of the integrated system requires substantial hardware resources, potentially limiting scalability for widespread deployment^73^. Additionally, the current framework focuses primarily on swimming applications, and further research is needed to validate generalizability across other sports domains.

Future research priorities encompass five concrete technical directions with quantifiable objectives and implementation timelines. First, computational efficiency optimization through neural architecture search (NAS) aims to develop lightweight policy networks that maintain 95% of current performance while reducing inference latency from 45ms to under 20ms and decreasing memory footprint by 60% (from 4.8GB to under 2GB per agent), enabling deployment on edge devices such as NVIDIA Jetson Xavier NX for pool-side real-time processing without cloud dependence, with prototype development targeted for Q2 2025 and validation across 500 + swimmer profiles by Q4 2025. Knowledge distillation techniques will compress the multi-agent ensemble into a single student model retaining 92% of teacher performance while achieving 5× speedup, reducing adaptation time for new swimmers from current 4.8 sessions to under 2 sessions. Second, federated meta-learning implementation will establish privacy-preserving cross-institutional collaboration where 10 + swimming programs contribute to a shared meta-model without exposing individual athlete data, implementing ε-differential privacy with ε = 0.5-1.0 providing formal privacy guarantees against membership inference attacks (success rate < 55% vs. 95% without protection), secure aggregation protocols using additive secret sharing to prevent central server access to local gradients, and blockchain-based audit trails ensuring transparent contribution tracking and proportional benefit allocation. A federated swimming benchmark dataset will be constructed by Q3 2025 comprising 1000 + anonymized swimmer profiles from NCAA Division I programs, international federations, and Olympic training centers, with standardized evaluation protocols measuring cross-site generalization, privacy-utility tradeoffs, and communication efficiency. Third, team sport extensions will adapt the multi-agent framework to water polo by modeling 7-player coordination through graph neural networks capturing spatial relationships and pass connectivity (implementation Q1-Q3 2026), synchronized swimming through multi-agent choreography optimization with aesthetic scoring functions balancing synchronization precision (temporal alignment within 50ms) and artistic expression quantified via pose diversity metrics (prototype by Q4 2025), and relay swimming by optimizing handover strategies through joint policy learning that minimizes transition time while maximizing individual leg performance (field testing in Q2 2026). Fourth, theoretical foundation advancement includes proving convergence guarantees for multi-agent meta-learning under non-stationarity by extending stochastic approximation theory to distributed settings with time-varying task distributions, establishing PAC-Bayesian generalization bounds for skill transfer that characterize sample complexity as a function of task relatedness and swimmer population diversity (manuscript submission targeted for ICML 2026), and analyzing reward shaping stability through Lyapunov-based methods that provide sufficient conditions for avoiding degenerate solutions in multi-objective optimization (publication goal: NeurIPS 2026). Fifth, practical deployment pathways involve pilot programs with 3 NCAA Division I teams (initiated Q1 2025) installing sensor infrastructure (8–12 synchronized 240fps underwater cameras at 2.5 m intervals, waterproof IMU arrays sampling at 1 kHz, edge computing nodes achieving < 50ms end-to-end latency), developing coaching dashboards with 3D biomechanical visualization and technique deviation heatmaps alongside athlete mobile applications providing immediate post-lap haptic feedback, and establishing commercial partnerships targeting subscription models at $49–99 monthly per athlete or $399–799 for team licenses with planned market entry in Q1 2026 focusing on collegiate programs, elite training centers, and Olympic development camps. Integration with existing timing systems (Omega, Daktronics) and meet management software (HyTek, Ares) will be completed by Q3 2025 to ensure seamless competition integration. Sixth, interdisciplinary extensions include sports physiology integration incorporating lactate threshold monitoring (via wearable biosensors) and VO2max modeling to optimize training intensity zones and periodization schedules (collaboration with University Sports Science Departments initiated Q2 2025), sports psychology modules addressing performance anxiety through biofeedback-guided relaxation training and mental imagery protocols embedded in the training loop (pilot testing with 50 athletes Q3 2025), and nutrition optimization using inverse reinforcement learning to infer personalized macronutrient requirements from training load and recovery patterns (algorithm development Q4 2025, clinical validation Q1 2026). Cross-domain transfer to related aquatic sports includes diving (rotational dynamics modeling and entry angle optimization using rigid body simulation, Q2-Q4 2025), open-water swimming (environmental adaptation for currents, waves, and navigation using domain randomization techniques, Q1-Q3 2026), and underwater hockey (3D spatial reasoning and team coordination in constrained environments, Q4 2026). Success metrics for these initiatives include: computational efficiency target of 20ms inference with 2GB memory by end-2025, federated learning deployment with 10 + institutions by mid-2026, team sport prototypes with 85% coach satisfaction by end-2026, theoretical papers accepted at top-tier ML conferences by 2026, and commercial pilots with 100 + athlete users generating $50K + monthly recurring revenue by Q2 2026. These specific, time-bound objectives with quantifiable deliverables provide a concrete research roadmap for advancing intelligent swimming training systems and expanding their impact across aquatic sports disciplines^74–76^.

## Data Availability

All data generated and analyzed during the current study are available from the corresponding author upon reasonable request.
